# Sustainable strategy for controlled phase formation of ZnO and ε-Zn(OH)_2_ nanoparticles for methylene blue degradation

**DOI:** 10.1039/d6na00515b

**Published:** 2026-08-06

**Authors:** Giuliana Taglieri, Valentina Maurizio, Matteo Cofini, Valeria Daniele, Claudia Mondelli, Orazio Spadaro

**Affiliations:** a Department of Industrial and Information Engineering and Economics, University of L'Aquila P.le E. Pontieri 1, Monteluco di Roio 67100 L'Aquila AQ Italy giuliana.taglieri@univaq.it; b SNAPTECH SRL, Spin-Off of the University of L'Aquila P.le E. Pontieri, Monteluco di Roio L'Aquila 67100 Italy; c CNR-IOM-OGG, Institut Laue Langevin 71 Avenue des Martyrs 38042 Grenoble CEDEX 9 France; d National Interuniversity Consortium of Materials Science and Technology, INSTM Via G. Giusti, 9 50121 Firenze Italy

## Abstract

This study reports a facile, one-step, and additive-free ion-exchange synthesis for the controlled formation of zinc oxide (ZnO) and zinc hydroxide (ε-Zn(OH)_2_) nanoparticles under room-temperature conditions with remarkably fast phase formation (within 2 minutes). The primary novelty lies in utilizing ordinary tap water as a green solvent, and an active chemical parameter to dictate phase evolution, thereby eliminating distilled water usage and energy-intensive thermal calcination. Structural and morphological characterization *via* XRD and TEM revealed a precise temperature-driven (15–45 °C) phase selection, yielding elongated ε-Zn(OH)_2_ structures at 15 °C, pure ZnO nanorods at 24–32 °C, and pseudo-spherical ZnO (9 nm) with a specific surface area of 35.76 m^2^ g^−1^ at 45 °C. Application of these nanomaterials in photocatalysis demonstrated exceptional efficiency, achieving over 99.2% methylene blue degradation under natural sunlight and 77% under indoor UV-A light (6 W lamp) within 150 minutes. Tauc plot analysis revealed a defect-induced bandgap narrowing, (*E*_g_ ≈ 3.21–3.27 eV *vs.* 3.27 eV for bulk ZnO), expanding its light-harvesting capacity into the solar spectrum. Crucially, kinetic and thermodynamic analyses correlated this performance with an anomalous negative apparent activation energy (*E*_ap_), revealing a non-Arrhenius regime governed by exothermic adsorption–desorption equilibria, typical of a Langmuir–Hinshelwood mechanism. Furthermore, post-reaction FTIR analysis confirmed the high structural stability and photocorrosion resistance of the catalysts. Overall, these findings highlight a highly sustainable, energy-efficient, and industrially scalable pathway for fabricating high-efficiency photocatalysts for environmental remediation.

## Abstract 2

This study reports an ultrafast, one-step, and additive-free ion-exchange synthesis enabling unprecedented room-temperature formation of ZnO and ε-Zn(OH)_2_ nanoparticles in just 2 minutes. Image partly generated with AI.

## Graphical abstract

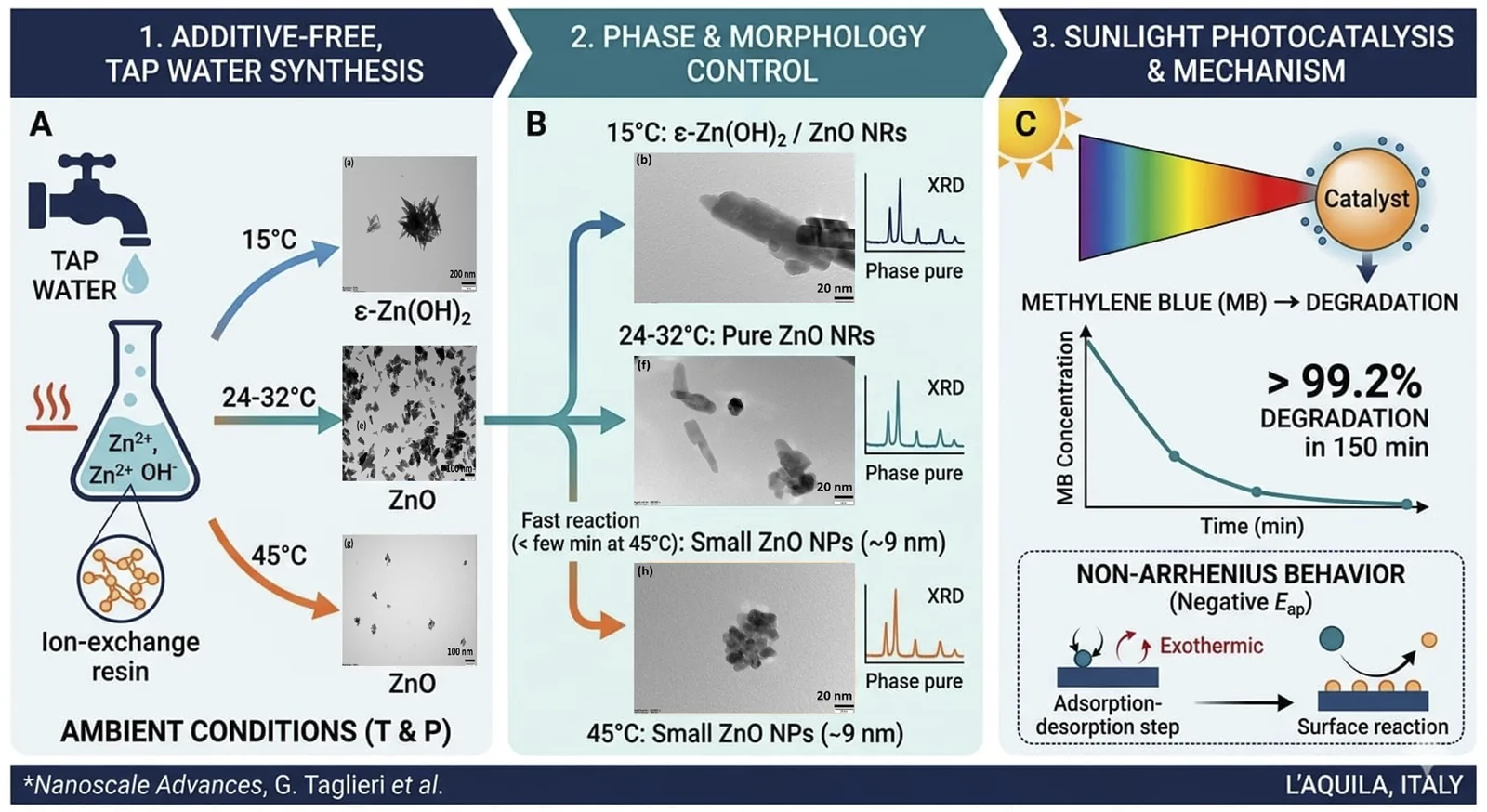

## 1 Introduction

Zinc-based nanomaterials have emerged as pivotal candidates for a diverse array of technological applications, owing to their unique combination of wide bandgap, potent UV absorption, photocatalytic activity, and piezoelectric properties.^1,2^ Beyond their functional versatility, the relative abundance, low cost, and minimal toxicity of zinc compounds align closely with the requirements for developing sustainable, multifunctional technologies. In particular, zinc oxide (ZnO) is extensively utilized as a high-performance semiconductor in fields ranging from photocatalytic pollutant degradation and water splitting^3–7^ to CO_2_ reduction, optoelectronics, and antimicrobial coatings.^8–15^ Beyond environmental remediation, ZnO and its related composites have attracted tremendous attention in cutting-edge fields due to their versatile electrochemical properties, finding widespread application in energy storage systems such as solar cells and high-performance supercapacitors.^16–20^ Furthermore, mixed ZnO/Zn(OH)_2_ nanostructures are gaining attention for their ability to integrate the semiconducting nature of ZnO with the high surface hydroxyl density of Zn(OH)_2_. These heterointerfaces facilitate superior charge separation and the generation of reactive oxygen species (ROS), significantly boosting photocatalytic efficiency.^21^

Despite their potential, the widespread adoption of these materials is often hindered by the limitations of traditional synthetic strategies. Conventional routes, such as sol–gel, hydrothermal, solvothermal, and precipitation methods, frequently require high temperatures, elevated pressures, multi-step procedures, or the addition of organic surfactants and complexing agents.^22–30^ While green synthesis *via* plant extracts offers an eco-friendly alternative,^31,32^ many of these processes still necessitate energy-intensive calcination to achieve the desired crystallinity and morphology. Such requirements raise concerns regarding scalability, cost-effectiveness, and environmental impact, underscoring the urgent need for simpler, low-energy synthetic alternatives.

The urgency of reducing climate impact has highlighted the necessity of developing green synthesis routes that operate under ambient conditions; however, additive-free, one-step methods remain scarcely reported.^33,34^ This work addresses this critical gap by introducing a low-energy, ion-exchange chemical synthesis of pure ZnO and ε-Zn(OH)_2_ nanoparticles operating at near-room temperature (15–45 °C) and atmospheric pressure. Crucially, rather than relying on high-purity media, this approach systematically evaluates the “tap water effect” not merely as a cost-effective, green solvent, but as an active chemical parameter that eliminates the energy-intensive production of distilled water while influencing phase evolution. Through subtle adjustments of the ambient synthesis temperature, this versatile and reproducible method enables precise, selective control over both phase composition (ε-Zn(OH)_2_*vs.* ZnO) and resulting morphologies (elongated rods *vs.* pseudo-spherical nanoparticles).

To demonstrate the practical utility of these nanomaterials, their photocatalytic activity was evaluated through the degradation of methylene blue (MB) under both solar and UV irradiation. Organic dyes like MB represent a significant environmental threat, with global production exceeding hundreds of thousands of tons annually.^35,36^ Due to their resistance to natural degradation and the inadequacy of traditional physical remediation techniques, which merely transfer pollutants from one phase to another without mineralization,^37–39^ advanced oxidation processes (AOPs) have become essential. In this regard, novel functional frameworks and composite materials are being extensively developed to optimize solar-driven treatments and synergistic pollutant degradation.^40–42^ Among these, heterogeneous photocatalysis using ZnO is particularly effective due to its favourable band structure for ROS production.^43–45^ Although the exceptional efficacy of ZnO-based catalysts for pollutant degradation – such as methylene blue (MB) – is extensively documented,^5,46–48^ most existing production methods remain energy-intensive. Therefore, achieving a fully predictable, cost-effective, and kinetically controlled synthesis of these pure phases without organic surfactants remains a crucial open challenge. This study shifts the paradigm of low-temperature zinc nanomaterials fabrication by addressing three interconnected scientific objectives. First, we provide a deep mechanistic insight into the temperature-driven phase evolution that dictates the sharp transition between ε-Zn(OH)_2_ and pure ZnO. Second, we unravel the chemical role of tap water constituents in facilitating rapid phase formation under ambient conditions within just 2 minutes. Finally, we correlate the exceptional photocatalytic performance of the synthesized nanostructures with an anomalous negative apparent activation energy (*E*_ap_), shedding light on a non-Arrhenius kinetic regime. This phenomenon, wherein the overall reaction rate decreases at elevated temperatures, is governed by exothermic adsorption–desorption equilibria of the dye molecules on the catalyst surface.^49^ Furthermore, defect-induced optical bandgap narrowing (*E*_g_ ≈ 3.21–3.27 eV *vs.* 3.37 eV for bulk-ZnO),^50^ justifies the superior harvesting capacity and photocatalytic performance under natural solar irradiation. Ultimately, this comprehensive structure–function-thermodynamic framework establishes a highly sustainable, industrially scalable, and energy-efficient pathway for environmental remediation.

## 2 Experimental

### 2.1 Synthesis of ε-Zn(OH)_2_/ZnO nanoparticles

#### 2.1.1 Materials and methods

Zinc chloride (ZnCl_2_, ≥98%), sodium hydroxide (NaOH, ≥98%), were purchased from Sigma-Aldrich and used as received. An anion-exchange resin in the Cl^−^ form (Marathon 550A, OH^−^, Dowex; nominal exchange capacity ≈ 1.1 eq L^−1^, average diameter 590 ± 50 µm according to the manufacturer) was used after conditioning. To evaluate the feasibility of an industrially scalable process and investigate the chemical influence of a real-water matrix on the phase evolution, ordinary tap water was systematically utilized as the reaction medium for all solutions, avoiding any distilled or deionized water pre-treatment.^51,52^

The resin was converted to the OH^−^ form and cleaned prior to use. In a typical preconditioning procedure, the resin (wet beads) was rinsed with 8 wt% NaOH solution (5× resin bed volume), then thoroughly washed with water until the washings were neutral (pH ∼7) and free of chloride. The preconditioned resin was stored in water and used as a wet slurry for synthesis. The amount of resin added was chosen to supply nominal OH^−^ equivalents of 1.1 per Zn^2+^.

For a solution volume *V*_sol_ (L), and ZnCl_2_ concentration *C*_Zn_ (mol L^−1^), eqn (1) is:

1 *n*_Zn^2+^_ = *C*_Zn_ × *V*_sol_

and the stoichiometric ratio between OH^−^ and Zn^2+^ moles is determined by eqn (2):

2 stoichiometric *n*_OH^−^_ = 2 × *C*_Zn_ × *V*_sol_

The OH^−^ equivalents supplied by the resin were calculated using eqn (3) as follows:

3 OH_resin_^−^ (mol) = *C*_resin_ (eq L^−1^) × *V*_resin_ (L)

where *C*_resin_ is the resin exchange capacity reported by the supplier. The resin volume *V*_resin_ was chosen to give the desired OH_resin_/OH_stoich_ ratio.

#### 2.1.2 Kinetics of nanoparticles formation: influence of temperature

To evaluate the influence of temperature on the phase formation and evolution within the tap water medium, four different controlled temperatures, all chosen around room temperature, were considered: 15 °C, 24 °C, 32 °C, 45 °C.

The samples synthesized were then labelled according to their synthesis temperature: Z15, Z24, Z32 and Z45.

All syntheses were performed at ambient pressure. The synthesis duration was adjusted according to the reaction temperature: experiments at 15 °C and 24 °C were performed for 180 minutes, whereas those at 32 °C and 45 °C were limited to 90 minutes. This difference reflects the need to adequately observe the stability and evolution of the phases formed.

A typical batch synthesis was carried out as follows. A ZnCl_2_ aqueous solution was prepared by dissolving a precise amount of precursor to achieve a concentration of 0.8 mol L^−1^ in tap water to a total volume of 100 mL. The preconditioned resin (wet beads) was added to the ZnCl_2_ solution under magnetic stirring (≈300 rpm). Resin volumes were adjusted to supply nominal OH^−^ equivalents. At the end of the synthesis, the resin beads were separated by a brief filtration by means of a sieve (aperture 200 µm) and regenerated with the NaOH solution to be ready for the next syntheses, according to a cyclic sustainable procedure. The obtained suspension was collected and, when required for subsequent solid-state characterization analyses (*e.g.*, XRD, TEM, and BET), dried in an oven at a temperature of 60 °C.

The formation kinetics of zinc oxide or hydroxide nanoparticles *via* ion-exchange was investigated for each temperature: 15 °C, 24 °C, 32 °C, and 45 °C. For this aim, aliquots were collected during the reaction process at 1, 2, 5, 15, 30, 45, 60, 90 minutes, and longer if necessary. Reaction progress was monitored by measuring the chlorides concentration (CC), by an ion-selective electrode (vernier), and pH values (Hanna Edge^pH^). Two parameters were defined to put in evidence the variation of chlorides concentration between two successive measurements: the rate, *R*, defined as the variation of the chlorides concentration over time, and the yield, *Y*, evaluated as follows by eqn (4):4
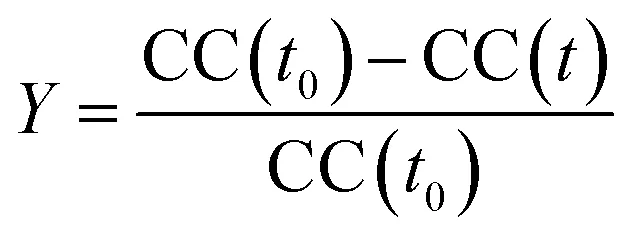


In parallel, we monitored the temporal evolution of the crystalline phases, in terms of crystal structure and phase composition, using X-ray diffraction (XRD). XRD analyses were carried out by using a PANalytical X'Pert PRO diffractometer equipped with Cu Kα radiation (*λ* = 1.5418 Å) operating at 40 kV and 40 mA, and a PIXcel1D X-ray detector. Powdered samples were mounted on zero-background sample holders and measurements were performed over a 2*θ* range of 5° to 70°, with a step size of 0.026° and a counting time of 200 seconds per step. Phase identification and profile analysis were carried out by HighScore Plus Software, referencing ICDD and ICSD international databases. Semi-quantitative phase analysis was carried out through Rietveld refinement, providing relative phase quantitative analysis and insights into the time- and temperature-dependent kinetics of phase formation. Diffraction data were further assessed by analysing peak shapes and widths, to estimate the average crystallite size using the Scherrer equation.^53^

#### 2.1.3 Structural and morphological characterization of ZnO and ε-Zn(OH)_2_

At the end of each synthesis, the obtained product was characterized in terms of phase composition, crystallinity, morphology, nanoparticle size, specific surface area (BET), and UV-visible absorbance response.

Phase composition and crystallinity were determined by X-ray diffraction (XRD), as previously described.

Surface functional groups and chemical bonding were analysed by Attenuated Total Reflection Fourier-Transform Infrared spectroscopy (ATR-FTIR Bruker Vertex 70v spectrometer). Spectra were recorded in the range of 4000–300 cm^−1^ with a resolution of 2 cm^−1^. ATR-FTIR analysis was used to identify characteristic vibrations of Zn–O, O–H groups, and residual precursor species, providing additional insight into phase formation.

Morphology, nanoparticle shape, and size distribution were investigated by transmission electron microscopy (TEM Philips CM100). For TEM observations, 0.1 mg of each sample was ultrasonically dispersed in ethanol (1 mL) for 30 min to obtain a homogeneous suspension. A drop of the suspension was then placed onto a carbon-coated copper grid and dried under ambient condition. Particle dimensions were determined by measuring the diameter or length, depending on the nanoparticle morphology (pseudospherical or rod-like), for at least 40 individual nanoparticles selected from multiple representative micrographs using ImageJ software. The measured values were then used to construct histograms from which the particle size distribution was obtained. Average particle dimension and standard deviation were calculated by fitting the experimental data with a Gaussian, log-normal, Weibull, or gamma distribution function and choosing the best fit according to the Akaike Information Criterion (AIC).^54^

The specific surface area was determined by nitrogen adsorption (BET method) using a Micromeritics ASAP2000 surface area analyzer. For each sample, about 0.6 g of dry powder are outgassed at 180 °C for 2 h prior to measurement. The BET surface area was calculated from the adsorption isotherm in the relative pressure range of 0.04 < *P*/*P*_0_ < 1.00.

### 2.2 Photocatalytic test

For the photocatalytic tests, methylene blue, MB, (C_16_H_24_ClN_3_O_3_S, 95%) purchased from Alquera has been used. Before starting the experiments, the MB solution with dispersed ZnO, was kept in the dark for 30 minutes to reach adsorption–desorption equilibrium. This step ensured that only photocatalytic activity was measured during the subsequent analysis. The residual MB concentration was determined spectrophotometrically at *λ* = 660 nm. The normalized concentration was defined as follows (eqn (5)):5
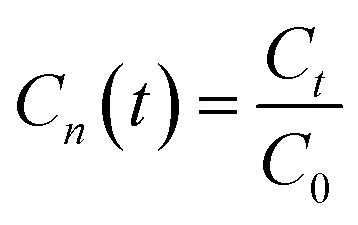
where *C*_*t*_ = *C*(*t*) and *C*_0_ = *C*(*t* = 0). The degradation efficiency has been calculated by the following equation (eqn (6)):6
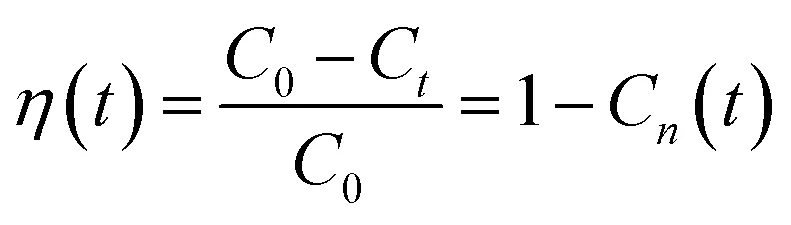


All tests under the sunlight were performed in triplicate and all tests under UV lamp were performed in duplicates.^75^

#### 2.2.1 MB degradation under sunlight

Owing to its semiconducting nature,^50,55^ ZnO nanoparticles synthesized at 24, 32, and 45 °C were systematically investigated for their photocatalytic performance in degrading MB under natural sunlight irradiation.

Degradation experiments of MB were performed using a 100 mL solution of 0.01 g L^−1^ MB and 1 g L^−1^ of ZnO (synthesized at 24, 32, and 45 °C) catalyst, maintained in dispersion by magnetic stirring throughout the entire experiment duration (150 min). The experiments were carried out in July between 10 : 00 and 13 : 00 in L'Aquila, Italy (42.35° N, 13.40° E), corresponding to the period of highest solar irradiation (about 800 W m^−2^). All experiments were performed under clear-sky conditions. The ambient temperature was approximately 30 °C, while the magnetic stirring plate reached about 47 °C due to solar irradiation, with no active temperature control. At 5′, 10′, 20′, 30′, 40′, 50′, 60′, 90′, 120′ and 150′ aliquots were withdrawn to investigate the degradation kinetics.

#### 2.2.2 MB degradation under UV lamp

Degradation experiments of MB by UV lamp were performed under the same condition as those used under sunlight.

The experiments were carried out indoors using a 365 nm UV lamp (VILBER LOURMAR VL-6.LC, 6 watt). The ambient temperature was 20 ± 0.1 °C, while the temperature of the magnetic stirring plate was actively controlled and set to 25 °C (ambient temperature), 32 °C (intermediate temperature), and 47 °C (same hot-plate temperature as the under-sunlight experiment) in separate experimental runs. At 5′, 10′, 20′, 30′, 40′, 50′, 60′, 90′, 120′, and 150′ aliquots were withdrawn to investigate the degradation kinetics.

#### 2.2.3 Kinetics and thermodynamics

Given that temperature is a key parameter affecting reaction kinetics, the temperature dependence of the process was systematically investigated. The influence of temperature on the apparent rate constant (*k*_ap_) was assessed using the Arrhenius equation^56^ (eqn (7)), which describes the relationship between *k*_ap_ and temperature as:7
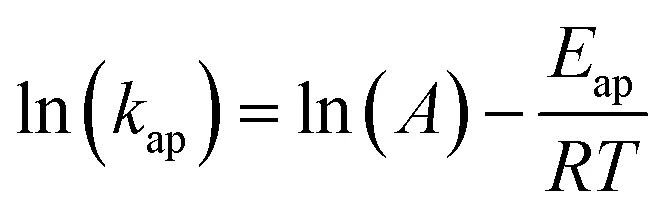
where *A* is a constant, *E*_ap_ is the apparent activation energy, and *R* is the universal gas constant. Furthermore, the temperature dependence of the rate constant was examined by applying the Eyring equation:^56^8
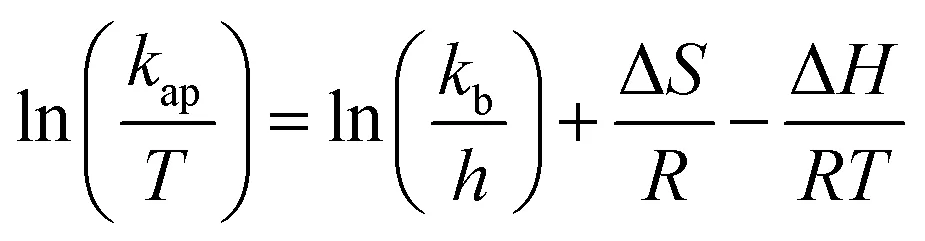


The rate constant (*k*_ap_) was determined by a pseudo-first order kinetic model,^57^ as shown in eqn (9) and (10):9
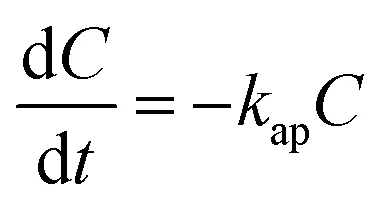
10
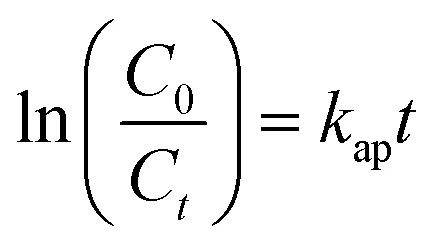


From these equations, the apparent activation energy (*E*_ap_), entropy change (Δ*S*), and enthalpy change (Δ*H*) were determined. Finally, the real intrinsic *E*_a_ was determined as follows:

11 *E*_a_ = *E*_ap_ − Δ*H*

#### 2.2.4 Data processing, absorption coefficient, and Tauc analysis

The results of the absorption spectra collected *via* spectrophotometer (transmission mode, from 800 nm to 200 nm) were used to determine the band gap energy of each of the ZnO nanoparticles using Tauc's equation (eqn (12)) for direct band gap material:^58^

12 (*αhν*)^2^ = *k*(*hν* − *E*_g_)

where *hν* is the photon energy, *E*_g_ is the band gap energy, *k* is a constant, and *α* is the absorption coefficient. The absorption coefficient *α*(*λ*) was estimated using the following relation (eqn (13)):13
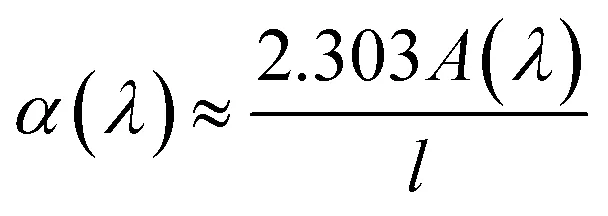
where *l* = 1.00 cm is the cuvette length and *A*(*λ*) is the absorbance registered for each wavelength value.

The optical bandgap energy was then determined by extrapolating the linear (steepest) region of the Tauc plot, (*αhν*)^*n*^*vs. hν*. The extrapolated line was extended to intersect the horizontal axis at (*αhν*)^*n*^ = 0, and the precise bandgap value is read at the interception.

## 3 Results

### 3.1 Kinetics of phase formation during ion-exchange synthesis

The variation of CC and pH values over synthesis time, providing information about the kinetics of the ion exchange processes, is shown in Fig. 1.

**Fig. 1:**
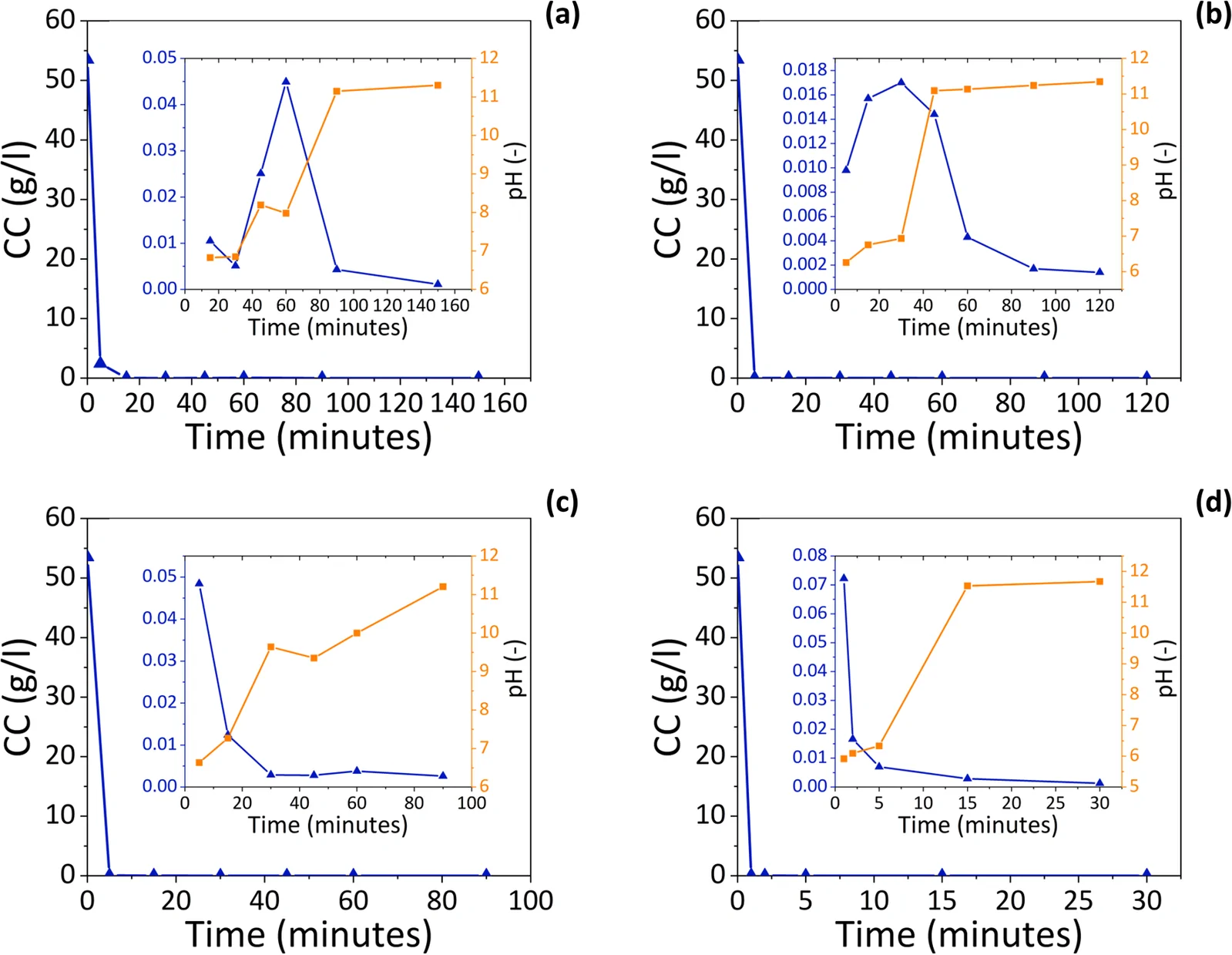
Variation of CC over synthesis time for: (a) Z15, (b) Z24, (c) Z32 and (d) Z45. In the insets: zoom images of the lowest CC values and pH values to put in evidence their mutual variations.

The corresponding numerical values have been reported in Table 1, together with *R* and *Y* values as well as the quantitative phase composition obtained by Rietveld refinement. These results reveal very fast kinetics at all investigated temperatures, characterized by a rapid increase of pH to alkaline values and a decrease in chloride concentration (CC) within the first minutes, followed by saturation stages with very low residual chloride levels. The chloride removal yield (*Y*) ranges from 95% after 5 minutes at 15 °C to 99% after only 1 minute at 45 °C.

**Table 1:** Synthesis parameters and kinetic evolution as a function of reaction time (*t*) at different temperatures: chloride concentrations (CC), pH, ion-exchange rate (*R*), yield (*Y*), and quantitative crystalline phases composition obtained *via* Rietveld refinement. Abbreviations: S = precursor phase (Simonkolleite); Z = Wurtzite ZnO; ε-ZH = ε- Zn(OH)_2_

Temp./time (*t*)	CC (g L^−1^)	pH	*R* (g L^−1^ min^−1^)	*Y* (%)	Crystalline phase composition (wt%)
**15 °C**					
0 min	53.5	5.348	—	—	—
15 min	0.0105	6.826	0.2	99.98	80.1 S – 19.9 Z
30 min	0.0051	6.848	3.6 × 10^−4^	99.99	82.5 S – 14.2 Z – 3.3 ε-ZH
45 min	0.0251	8.194	−1.3 × 10^−3^	99.95	72.6 S – 15.3 Z – 12.1 ε-ZH
60 min	0.0449	7.980	−1.3 × 10^−3^	99.92	37.6 S – 8.4 Z – 54.0 ε-ZH
90 min	0.0043	11.149	1.3 × 10^−3^	99.99	14.7 Z – 85.3 ε-ZH
150 min	0.0011	11.302	5.3 × 10^−5^	100	9.7 Z – 90.3 ε-ZH

**24 °C**					
0 min	53.5	5.348	—	—	—
5 min	0.0098	6.258	10.7	99.98	70.2 S – 29.8 Z
15 min	0.0157	6.754	−5.9 × 10^−4^	99.97	52.8 S – 47.2 Z
30 min	0.0170	6.936	−8.7 × 10^−5^	99.97	40.8 S – 57.7 Z – 1.5 ε-ZH
45 min	0.0144	11.093	1.7 × 10^−4^	99.98	9.6 S – 88.4 Z – 2.0 ε-ZH
60 min	0.0043	11.135	6.7 × 10^−4^	99.99	2.3 S – 96.9 Z – 0.8 ε-ZH
90 min	0.0017	11.239	8.7 × 10^−5^	100	99.1 Z – 0.9 ε-ZH
120 min	0.0014	11.342	0.1 × 10^−6^	100	99.4 Z – 0.6 ε-ZH

**32 °C**					
0 min	53.5	5.348	—	—	—
5 min	0.0484	6.634	10.7	99.91	74.9 S – 25.1 Z
15 min	0.0124	7.270	3.6 × 10^−3^	99.98	99.8 Z – 0.2 ε-ZH
30 min	0.0029	9.641	6.3 × 10^−4^	100	100 Z
45 min	0.0028	9.351	6.7 × 10^−6^	100	100 Z
60 min	0.0038	10.000	6.7 × 10^−5^	100	100 Z
90 min	0.0026	11.205	0.4 × 10^−5^	100	100% Z

**45 °C**					
0 min	53.5	5.348	—	—	—
1 min	0.0722	5.919	53.4	99.87	15.4 S – 84.6 Z
2 min	0.0166	6.094	5.6 × 10^−2^	99.97	100 Z
5 min	0.0070	6.336	3.2 × 10^−3^	99.99	100 Z
15 min	0.0029	11.532	4.1 × 10^−4^	100	100 Z
30 min	0.0013	11.670	1.1 × 10^−4^	100	100 Z

Starting from an initial concentration of 58.67 g L^−1^, the chloride concentration decreases to approximately 2.45 g L^−1^ after 5 minutes at 15 °C and to 0.07 g L^−1^ after 1 minute at 45 °C, with comparable saturation values reached at the end of the syntheses (0.0011 g L^−1^ and 0.0013 g L^−1^, respectively).

Examining the behaviour at each temperature, at 15 °C the reaction proceeded relatively slowly in the early stage together with a neutral pH. After 15 minutes, a rapid decrease in CC and a corresponding increase in pH at 11.15 was observed. After 45 minutes, a slight increase in CC and decrease in pH were observed, before both parameters stabilised at longer times.

At 24 °C, the decrease in CC was slightly faster and more pronounced, and a pH of 11 was rapidly reached after 5 minutes. As seen at 15 °C, after 30 minutes a slight CC increase was observed together with a small decrease in the pH value (see the curve in the inset), and they decreased and increased progressively over time.

In the synthesis carried out at 32 °C, the decrease in CC and the corresponding increase in pH occurred even more pronouncedly and without oscillations over time. Stabilisation of both parameters was reached after approximately 30 minutes.

Finally, at 45 °C, the ion-exchange proceeded with remarkable speed: the CC decreased sharply within the first minute, indicating an extremely rapid exchange between Cl^−^ and OH^−^ in the batch. The pH increased accordingly, rising from its initial value (pH = 4,7) to stable values around 11.5, reached shortly after only 2 minutes. Both CC and pH remained constant for the remainder of the reaction, suggesting that equilibrium was achieved very rapidly.

Overall, the data indicate a clear correlation between chloride removal and pH across all synthesis temperatures. Higher temperatures promote a faster CC decrease and a quicker stabilization of pH, while lower temperatures delay these effects due to slower ion-exchange kinetics.

In parallel, the analysis carried out by means of X-ray diffraction allowed for relating the solid phase formation to the variation of CC and pH values measured at different times. Fig. 2 shows the XRD patterns of the samples synthesized at different temperatures, following the kinetics from the beginning to the end of the synthesis reaction.

**Fig. 2:**
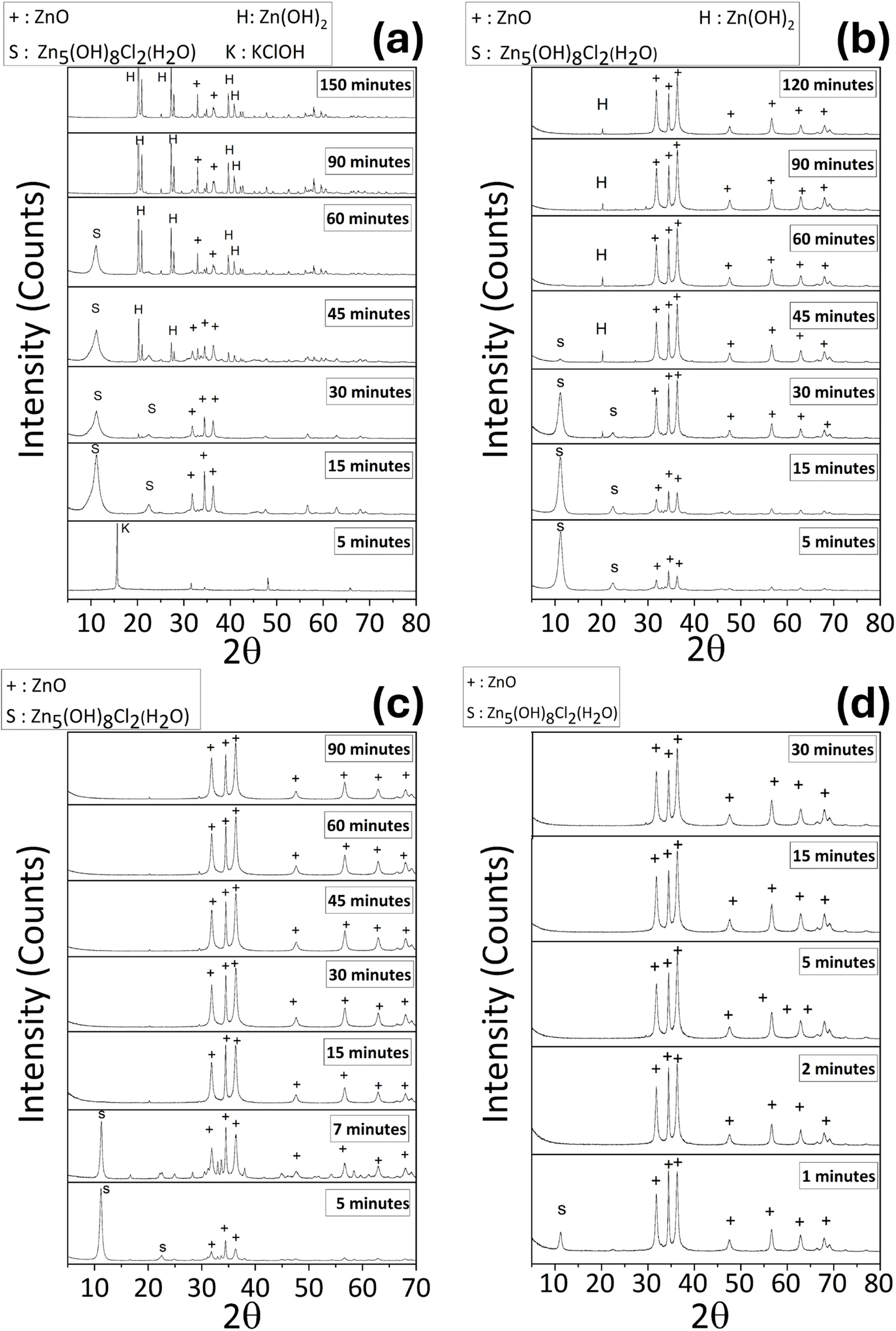
XRD patterns of synthesis at: (a) 15 °C, (b) 24 °C, (c) 32 °C and (d) 45 °C. Legend and peak indexing: (+) ZnO: (010) at 31.8°, (002) at 34.3°, (011) at 36.2°, (012) at 47.4°, (110) at 56.6°, (013) at 62.8°, (112) at 67.9° 2*θ* (ICSD #98-005-7478); (H) ε-Zn(OH)_2_: main characteristic reflections at (011) 20.2°, (101) 20.9°, (111) 27.2°, (102) 27.8° 2*θ* (ICSD #98-005-0447); §: Zn_5_(OH)_8_Cl_2_ H_2_O: main peak at (003) at 11.2° 2*θ* (ICSD #98-003-4904).

Starting with the lowest synthesis temperature, 15 °C, after 5 minutes, in the dried powders only an initial phase of zinc chloride hydroxide – beta (KClOH, ICSD pattern #98-001-5900) is observed, together with a small signal, at 11 220°2*θ*, attributable to the main Bragg peak of Simonkolleite (Zn_5_(OH)_8_Cl_2_·(H_2_O), ICSD pattern #98-003-4904), known as a precursor in the ZnO formation, both by thermal annealing at 500 °C or at lower temperature processes.^34^ After 15 minutes, KClOH disappears, and Simonkolleite notably increases, reaching a relatively quantitative percentage of 80.1%. The formation of the hexagonal Wurtzite structure of zinc oxide (ZnO) (ICSD pattern #98-005-7478) is also observed, having a relative quantitative percentage of 19.9%. Although the perfect matching with the Wurtzite structure, the newly formed ZnO shows a definite orientation of the (002) Bragg peak (at 34 432°2*θ*), in contrast to the main standard Wurtzite (001) Bragg peak (011) (placed at 36 225°2*θ*). After 30 minutes, we observe an initial formation of zinc hydroxide (ε-Zn(OH)_2_, ICSD pattern #98-005-0447, revealed from the appearance of its (011) main Bragg peak, at 20 182°2*θ*. After 45 minutes, Simonkolleite and ZnO tend to decrease, in favour of the ε-Zn(OH)_2_ development, which maintains a crystalline orientation on the main Bragg peak. Increasing time, after 90 minutes, ε-Zn(OH)_2_ remains the main phase present until the end of the synthesis, where the relative percentage of zinc hydroxide and zinc oxide reaches an equilibrium of about 7 : 1.

At 24 °C, already after 5 minutes, we observe both Simonkolleite and ZnO, with relative quantities of about 70% and 30%, respectively; moreover, as occurred at 15 °C, ZnO exhibits the orientation along the (002) Bragg peak. With increasing time, Simonkolleite progressively decreases until 45 minutes, and, correspondingly, ZnO increases, reestablishing the (011) peak as the main peak and destroying the orientation after 120 minutes. Additionally, weak reflections associated with ε-Zn(OH)_2_ appear after 30 minutes, likely due to slight acidification, which remains almost stable since the end of the synthesis.

At 32 °C, a similar phase composition is observed after 5 minutes, but Simonkolleite rapidly disappears with increasing time, and it is fully transformed into ZnO Wurtzite hexagonal phase already after 15 min. After 30 minutes, ZnO remains the only phase, with an orientation along the (002) Bragg peak, which is less pronounced over time.

At 45 °C the kinetics are extremely rapid, and Simonkolleite fully transforms into ZnO already after 2 minutes. Also in this case, an initial orientation along the (002) Bragg peak is observed at early stages, which decreases with the synthesis time.

The structural properties (lattice constants and cell volumes) and the crystallite sizes determined using Scherrer's formula are reported in Table 2 and 3, respectively.

**Table 2:** Structural properties (lattice parameters *a*, *b*, *c*, cell volume *V*, and *c*/*a* ratio) and Rietveld refinement residuals of ε-Zn(OH)_2_ and ZnO nanoparticles, obtained by Rietveld analyses at different synthesis temperatures and reaction times

Temp./time (*t*)	Phase	*D*_(*hkl*)_ values			
**15 °C**	**ε-Zn(OH)**_**2**_	***D***_**(011)**_ **(nm)**	***D***_**(101)**_ **(nm)**	***D***_**(111)**_ **(nm)**	**Average *D***_***hkl***_ **(nm)**
30 min		78.5	74.2	49.2	67.3
45 min		80.6	80.2	76.4	79.1
60 min		196.0	195.0	184.7	191.9
90 min		175.8	172.8	150.8	166.5
120 min		127.3	126.8	135.9	130.0
150 min		175.3	175.2	172.4	174.3

**24 °C**	**ZnO**	***D***_**(100)**_ **(nm)**	***D***_**(002)**_ **(nm)**	***D***_**(011)**_ **(nm)**	**Average *D***_***hkl***_ **(nm)**
5 min		24.0	22.3	21.2	22.5
15 min		18.8	18.1	17.6	18.2
30 min		19.5	18.7	18.2	18.8
45 min		17.4	16.9	16.6	17.0
60 min		16.9	16.4	16.1	16.5
90 min		15.4	15.2	15.1	15.2
120 min		16.6	16.1	15.8	16.2

**32 °C**	**ZnO**	***D***_**(100)**_ **(nm)**	***D***_**(002)**_ **(nm)**	***D***_**(011)**_ **(nm)**	**Average *D***_***hkl***_ **(nm)**
5 min		17.2	16.0	15.3	16.2
15 min		16.2	15.3	14.9	15.5
30 min		13.0	34.7*	12.0	19.9
45 min		13.4	34.6*	12.0	20.0
60 min		12.8	35.2*	12.1	20.0
90 min		13.2	37.0*	12.6	20.0

**45 °C**	**ZnO**	***D***_**(100)**_ **(nm)**	***D***_**(002)**_ **(nm)**	***D***_**(011)**_ **(nm)**	**Average *D***_***hkl***_ **(nm)**
1 min		16.4	15.6	15.2	15.7
2 min		13.0	31.5	12.5	19.0
5 min		12.0	23.4	10.6	15.3
15 min		11.7	23.2	10.7	15.2
30 min		12.3	22.4	11.7	15.4

**Table 3:** Microstructural crystallite sizes (*D*_*hkl*_) calculated by the Scherrer formula for each synthesis temperature and phase, in relation to the corresponding (*hkl*) crystallographic plane

Temp./time	Phase	*a* (Å)	*b* (Å)	*c* (Å)	*c*/*a*	*V* (Å^3^)
**15 °C**	**ε-Zn(OH)**_**2**_					
30 min		4.903	5.139	8.462	—	213.19
45 min		4.908	5.147	8.475	—	214.12
60 min		4.910	5.148	8.475	—	214.23
90 min		4.910	5.147	8.475	—	214.21
120 min		4.908	5.147	8.474	—	214.12
150 min		4.909	5.147	8.474	—	214.13

**24 °C**	**ZnO**					
5 min		3.251	—	5.209	1.602	47.676
15 min		3.247	—	5.203	1.602	47.508
30 min		3.250	—	5.208	1.602	47.657
45 min		3.251	—	5.209	1.602	47.679
60 min		3.251	—	5.209	1.602	47.671
90 min		3.201	—	5.209	1.628	47.669
120 min		3.250	—	5.209	1.602	47.652

**32 °C**	**ZnO**					
5 min		3.247	—	5.206	1.603	47.548
15 min		3.250	—	5.209	1.603	47.642
30 min		3.251	—	5.211	1.603	47.683
45 min		3.251	—	5.210	1.603	47.671
60 min		3.251	—	5.211	1.603	47.682
90 min		3.250	—	5.209	1.603	47.651

**45 °C**	**ZnO**					
1 min		3.252	—	5.212	1.603	47.722
2 min		3.251	—	5.211	1.603	47.701
5 min		3.251	—	5.212	1.603	47.714
15 min		3.251	—	5.212	1.603	47.714
30 min		3.251	—	5.212	1.603	47.717

These parameters were precisely evaluated *via* full-profile Rietveld refinement, yielding highly satisfactory quality factors across all analyzed conditions, with a Goodness-of-Fit (GoF) index around 2.0. Such residual values are standard and highly representative for nanostructured systems, where nanometre-scale grain boundaries, local lattice strains, and significant peak broadening inherently affect the profile fitting compared to coarse bulk crystals without compromising the accuracy of the structural determination.^59^

Regarding the structural analysis carried out on the ε-Zn(OH)_2_ synthesized at 15 °C, a slight increase in lattice constants is observed as the synthesis time increases up to 60 min, after which the values tend to stabilize. Parallelly, crystal size analysis indicates that crystal growth initially occurs preferentially along the (011) and (101) planes, as evidenced by the *D*_111_ values being lower than *D*_001_ and *D*_101_ up to 30 min. From 60 min onward, the *D*_111_ values increase, and crystal sizes become comparable along all three crystallographic planes of the orthorhombic structure, suggesting a more equiaxed growth regime.

From the measurements of the structural parameters, it was observed that the hexagonal ZnO unit cell expands as the reaction proceeds from 24 °C up to 45 °C, followed by a slight contraction at longer reaction times. Consistently, the *c*/*a* ratio shows a small but progressive increase with increasing reaction time. The microstructural analysis shows that, for all temperatures, ZnO crystal sizes are significantly smaller than those measured for ε-Zn(OH)_2_ and indicate a tendency in equiaxed growth along the three main crystallographic planes at very early stages. Then, *D*_*hkl*_ values exhibit a slight decrease with increasing reaction time as well as upon increasing the synthesis temperature at room temperature. However, except for the samples obtained at 24 °C, a pronounced preferential growth along the (002) plane becomes evident at 32 °C and 45 °C. Specifically, this preferential growth particularly occurs at 32 °C after 30 min, this anisotropy persists until the end of the synthesis. If we consider the average *D*_*hkl*_ values at different times, they range from 16.2 nm, 20.0 nm to 15.4 nm, at 24 °C, 32 °C, and 45 °C, respectively, denoting a slight reduction with increasing temperature probably attributed to the highest kinetics. The highest value measured for the intermediate temperature is attributed to the anisotropy associated with the plane (002).

These results highlight the extraordinary ability of the proposed synthesis method in assuring, also after a few minutes from the beginning of the synthesis, at room temperature or in a restricted range around it, the possibility of fast and pure formation of ZnO characterized by very low crystallite size. In addition, by a slight lowering of the temperature, the formation of almost pure nanostructured ε-Zn(OH)_2_ is easily tuned in a rapid process.

### 3.2 Transmission electron microscopy (TEM)

In agreement with the XRD results, representative transmission electron microscopy (TEM) images are presented in Fig. 3. The corresponding histograms, illustrating the statistical particle size distributions for each synthesis temperature, are shown in Fig. 4, where depending on particle morphology, either length (*L*) or diameter (*D*) was used to determine the distribution. Solid lines represent the best–fit curves to the data.

**Fig. 3:**
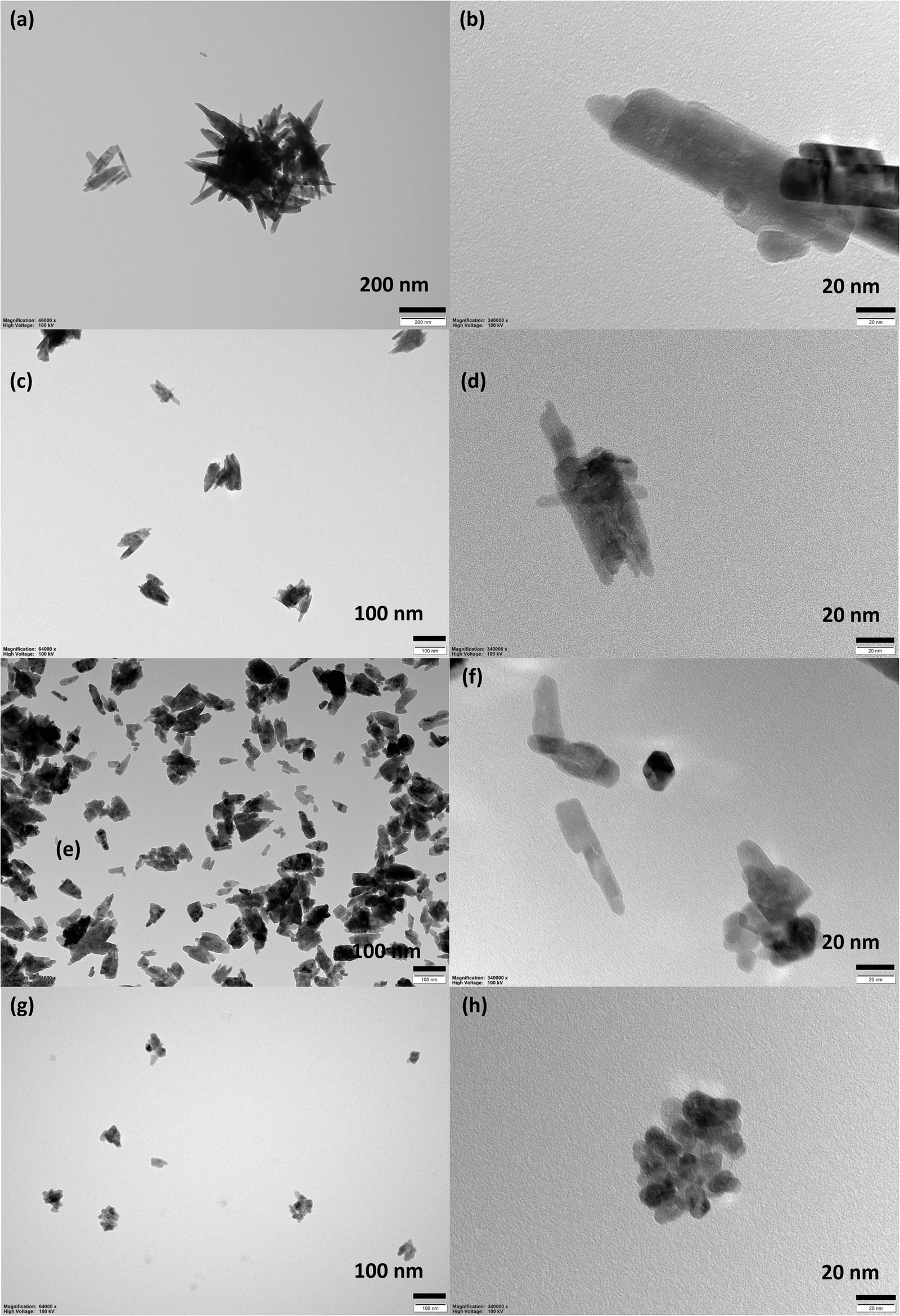
Representative TEM images of the particles at different temperature of synthesis, at low magnification (64.000×, on the left) and high magnification (450.000×, on the right): (a and b) 15 °C; (c and d) 24 °C; (e and f) 32 °C; (g and h) 45 °C.

**Fig. 4:**
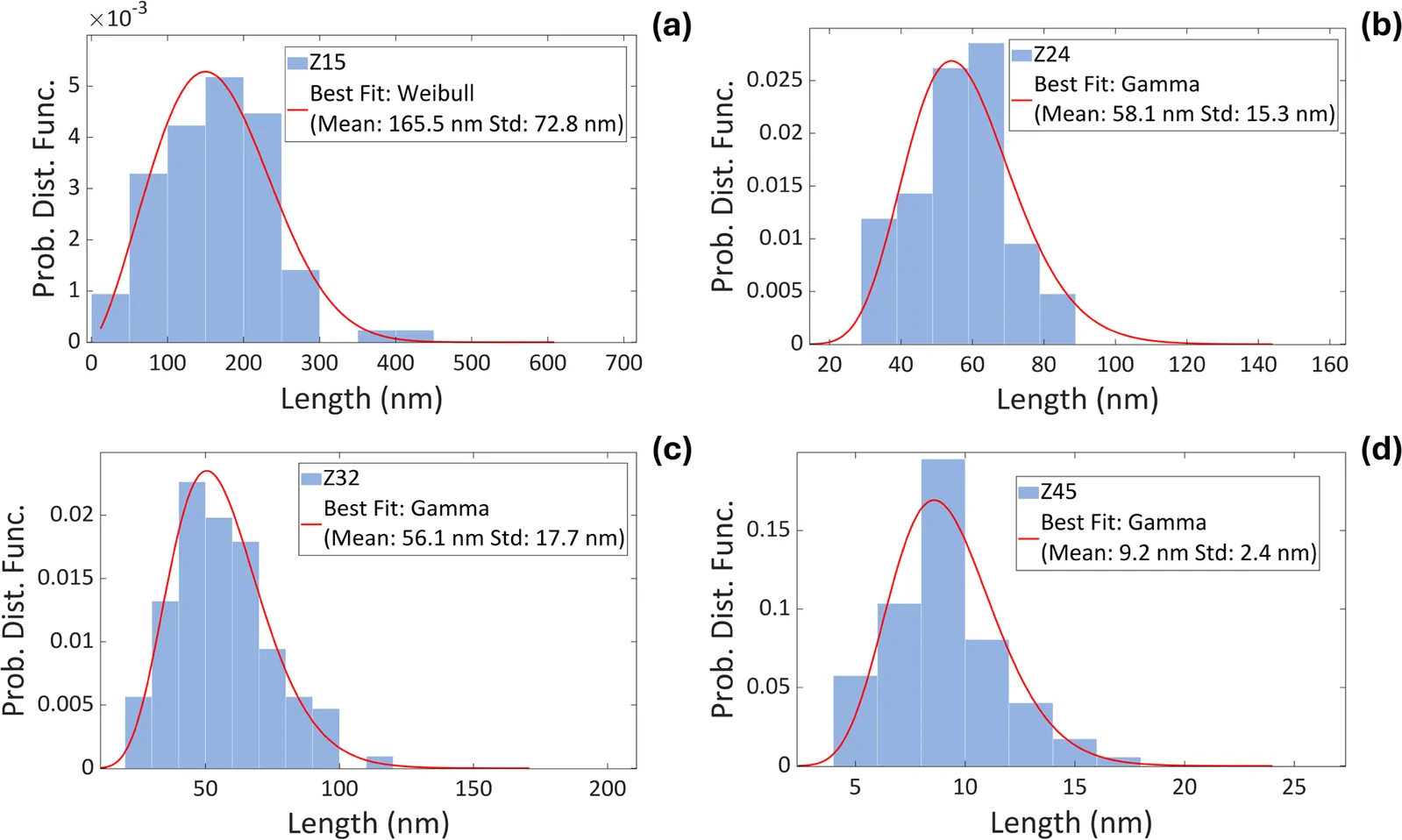
Size distributions obtained from TEM images 3of the nanoparticles synthesised at different temperatures. (a) Z15 (Weibull), (b) Z24 (gamma), (c) Z32 (gamma), and (d) Z45 (gamma).

TEM analysis revealed a profound temperature-dependent transformation in both the chemical nature and the physical habit of the nanostructures. This evolution is closely coupled with the phase transitions observed *via* XRD, moving from a multi-phase hydroxide system to phase-pure Wurtzite ZnO.

At the lowest synthesis temperature (15 °C), the morphology is dominated by elongated, pencil-like structures. The particle length follows a Weibull distribution (<*L*> = 166 ± 73 nm), with a high standard deviation reflecting a polydisperse system. In early-stage precipitation, Weibull behavior typically signifies a stochastic growth process in which a random distribution of structural defects and non-uniform supersaturation levels^60,61^ govern the rate. This result aligned with the XRD kinetics analysis showing a slow, competitive formation of different hydroxide phases before reaching a 7 : 1 equilibrium with ZnO.

Increasing the temperature to 24 °C and 32 °C marks the emergence of crystalline ZnO. A rod-like morphology is observed, and the particles undergo significant contraction (<*L*> = 58 ± 15 nm and <*L*> = 56 ± 18 nm). At 24 °C, the observation of small agglomerates suggests the onset of oriented attachment, where primary nanocrystals fuse along preferential crystallographic directions to minimize surface energy.^62^ This is supported by XRD results showing a pronounced (002) orientation in the early stages. The transition to a gamma distribution at these temperatures indicates a shift toward a more regulated “nucleation-and-growth” regime.^63^ Interestingly, at 32 °C, while XRD reveals a significant crystallographic anisotropy (*D*_002_ > *D*_100_), TEM shows the occasional appearance of hexagonal-shaped nanoparticles. This suggests that as the kinetics accelerate (with Simonkolleite transforming into ZnO in just 15 minutes), the system favors better-defined crystalline facets over the initial elongated habit.

At 45 °C, morphology evolves into pseudo-spherical nanoparticles with a sharply reduced average length (<*L*> = 9 ± 2 nm). This dramatic shift is a hallmark of the LaMer burst nucleation mechanism: the higher thermal energy exponentially increases the nucleation rate, leading to a massive generation of nuclei that rapidly depletes the zinc precursors.^64^ A noteworthy observation is the apparent decoupling between the TEM-observed morphology (pseudo-spherical) and the XRD-calculated crystallite anisotropy (*D*_002_ ≫ *D*_100_). This suggests that even if the underlying crystalline domains grow preferentially along the *c*-axis, the extreme transformation kinetics at 45 °C, where the Simonkolleite-to-ZnO conversion is nearly instantaneous (within 2 minutes), prevent the development of well-defined elongated facets. Instead, the particles adopt a compact, equiaxed shape to minimize the surface-to-volume ratio in a kinetically dominated environment.^65^

### 3.3 BET analysis

The nitrogen adsorption–desorption isotherms of samples synthesized at different temperatures (15–45 °C) are shown in Fig. 5.

**Fig. 5:**
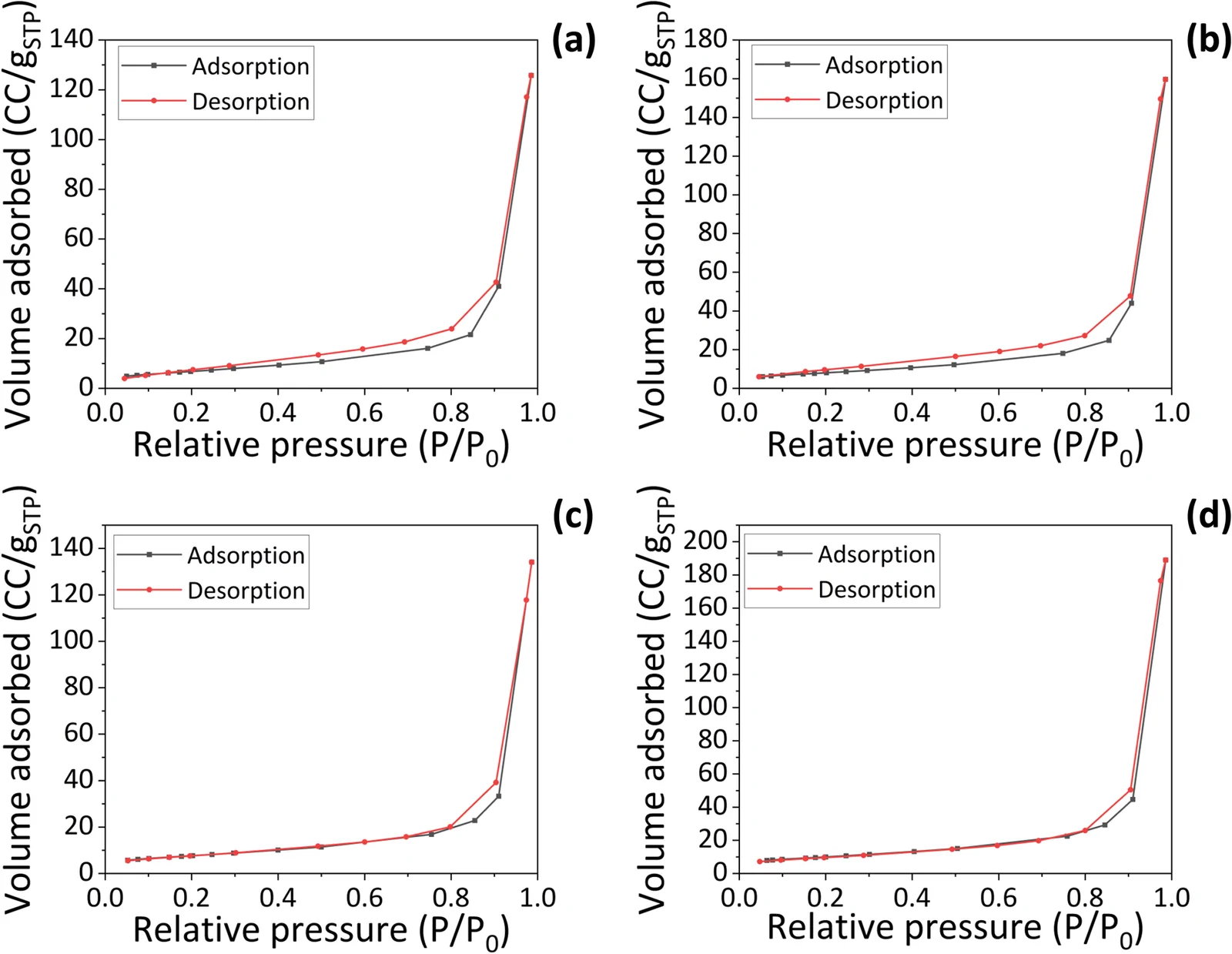
Adsorption–desorption isotherms of samples synthesized at (a) 15 °C, (b) 24 °C, (c) 32 °C and (d) 45 °C.

The specific surface area, determined by the BET method, increases progressively with synthesis temperature, from 24.78 m^2^ g^−1^ at 15 °C to 28.93 m^2^ g^−1^ at 24 °C, 30.09 m^2^ g^−1^ at 32 °C, and 35.76 m^2^ g^−1^ at 45 °C. This trend is in excellent agreement with the temperature-driven morphological evolution observed by TEM. At 15 °C, the relatively low surface area reflects the presence of elongated, pencil-like particles with limited accessible surface. Increasing the temperature to 24 °C and 32 °C leads to the formation of smaller rod-like ZnO nanostructures and a corresponding increase in surface area, consistent with a transition toward a more uniform nucleation-and-growth regime. At 45 °C, the highest BET surface area is achieved, in line with the formation of pseudo-spherical nanoparticles with significantly reduced dimensions.

All samples exhibit type IV isotherms according to the IUPAC classification, indicating the presence of mesoporosity. The adsorption branch shows a sharp increase at high relative pressures (*P*/*P*_0_ > 0.8), suggesting that capillary condensation predominantly occurs within interparticle voids rather than intrinsic pores. The isotherms are characterized by a well-defined but relatively narrow hysteresis loop, indicative of limited mesoporosity mainly associated with interparticle packing. The shape of the hysteresis loop is consistent with a type H3 hysteresis, typically associated with aggregates of non-rigid particles forming slit-like pores,^66^ supporting the hypothesis that the porosity originates from the assembly of nanostructures rather than from an intrinsic porous framework.

A progressive narrowing of the hysteresis loop is observed with increasing synthesis temperature, becoming almost negligible at 45 °C. This behaviour can be interpreted within the framework of the LaMer nucleation mechanism. At higher temperatures, rapid burst nucleation leads to the formation of a high density of small, quasi-spherical nanoparticles, limiting anisotropic growth and reducing the development of extended interparticle voids. Physicochemically, the narrowness of the loops highlights the fundamentally non-porous nature of the individual crystalline entities (ultra-small ε-Zn(OH)_2_, nanorods and pseudo-spherical ZnO). The resulting porosity is strictly textural, arising from open, shallow interparticle interstitial spaces where nitrogen condensation and evaporation occur with minimal thermodynamic restrictions or cavitation effects. Consequently, although the specific surface area increases, the mesoporosity becomes less pronounced, reflecting a more compact nanoparticle assembly. The absence of significant uptake at low relative pressures further confirms the negligible contribution of microporosity.

Overall, the adsorption–desorption behaviour closely reflects the temperature-dependent structural evolution of the system, with surface area and mesoporosity primarily governed by particle size reduction and aggregation mode. Such textural features are particularly favourable for photocatalytic applications, as they facilitate mass transport and increase the availability of active surface sites.

### 3.4 MB photocatalytic degradation

#### 3.4.1 Under sunlight

The ZnO synthesis at 24, 32, and 45 °C shows an interesting potential as catalysts for MB degradation under sunlight. In Fig. 6 the normalized MB concentration and efficiencies over time have been reported.

**Fig. 6:**
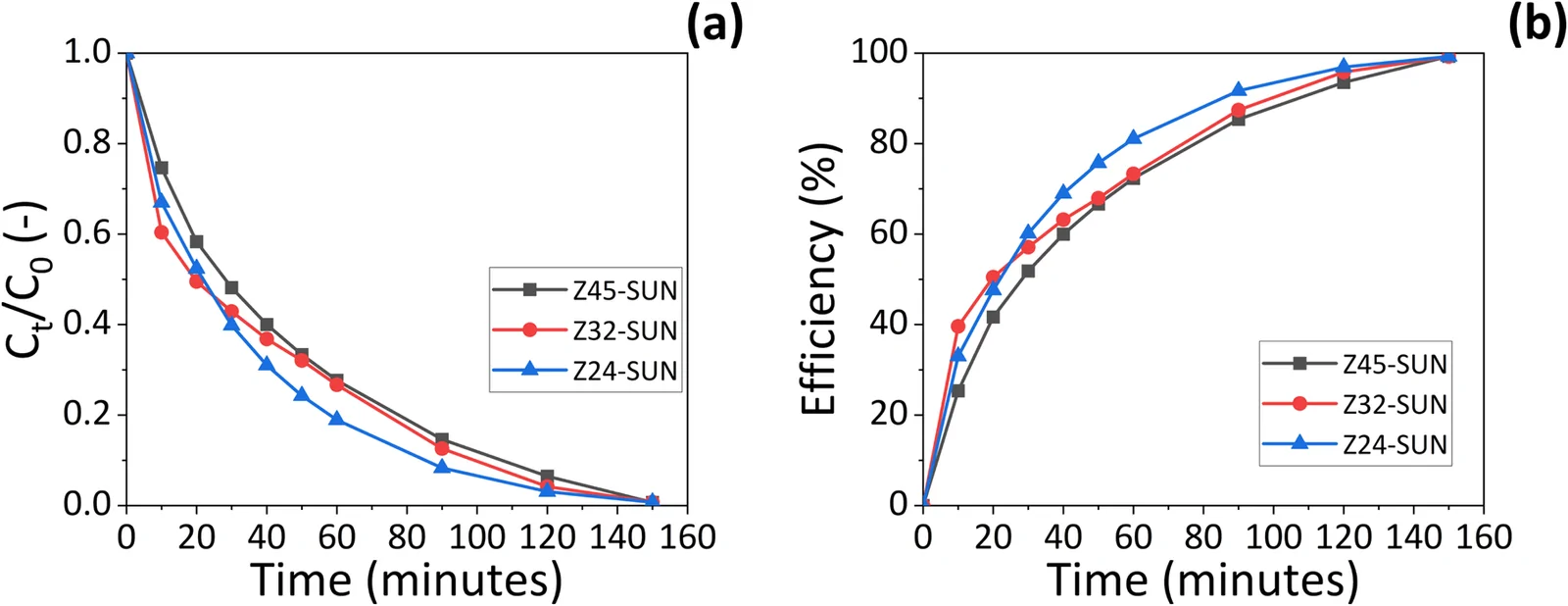
(a) Normalized MB concentration and (b) efficiencies under natural solar irradiation.

At 150′, all the samples reach an MB removal efficiency higher than 99.2%. In general, the samples do not exhibit significantly different behaviour from one another.

#### 3.4.2 Under 365 nm UV lamp

The catalytic performance of the Z45 and Z24 samples was further investigated under UV light (*λ* = 365 nm) at different operating temperatures (25, 32 and 47 °C). The results have been reported in Fig. 7. For both samples, a clear temperature-dependent trend was observed: the photocatalytic efficiency consistently decreased as the operating temperature increased. Specifically, the highest degradation rate was achieved for the Z45 sample at 25 °C, followed by the tests conducted at 32 °C and 47 °C. A similar performance hierarchy with respect to temperature was exhibited by the Z24 sample, confirming the systematic nature of this effect.

**Fig. 7:**
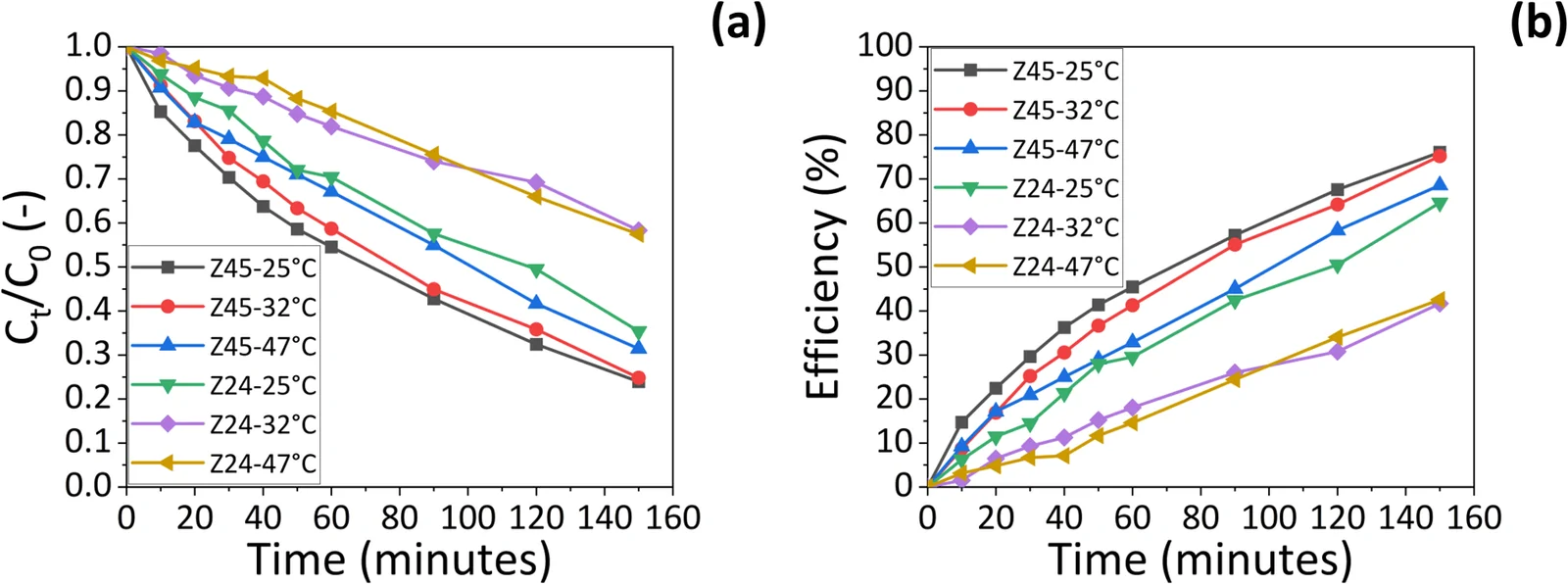
(a) Normalized MB concentration and (b) removal efficiencies under 365 nm UV-lamp at different temperatures (25 °C, 32 °C and 47 °C) for Z24 and Z45 samples.

Overall, Z45 demonstrated superior photocatalytic activity compared to Z24 across all tested conditions, which can be primarily attributed to its larger specific surface area providing a higher density of active adsorption sites.^67^

The observed decline in photocatalytic degradation rates at elevated temperatures indicates that the process is not governed by a thermally activated elementary step. Instead, higher thermal energy increases molecular disorder at the catalyst–solution interface, thereby impeding the dynamic adsorption/photocatalysis/desorption sequence.^68^ Consequently, the overall process appears to be controlled by adsorption–desorption equilibria and enhanced charge carrier recombination at higher temperatures.^67,68^

To contextualize the photocatalytic efficiency of the synthesized materials, the performance of sample Z45 was compared with recently reported pure, un-doped ZnO catalysts evaluated for methylene blue (MB) degradation under both natural solar irradiation and artificial UV/visible light sources (Table 4). Under natural sunlight, Z45 achieves an outstanding degradation efficiency of 99.2% within 150 min using a catalyst loading of 1.0 g L^−1^. While a comparable complete degradation (100% in 180 min) was reported for sol–gel synthesized ZnO,^69^ that system required double the catalyst dosage (2.0 g L^−1^) and an energy-intensive calcination step at 500 °C for 2 h. Similarly, ZnO prepared *via* mechanochemical synthesis followed by calcination at 500 °C yielded a significantly lower degradation efficiency (68.70% at 60 min).^70^ Under monochromatic indoor UV-A illumination (365 nm, 6 W), Z45 displays a competitive degradation efficiency of 77.0% in 150 min. Notably, this performance surpasses that of ZnO prepared *via* high-pressure sol–gel and calcined at 700 °C for 4 h, which achieved only 72.3% degradation after an extended irradiation time of 420 min under a higher lamp power (8 W).^71^ Although higher efficiencies under artificial light are reported in some literature works,^72,73^ these are routinely achieved by using lower initial dye concentrations (5 ppm)^72^ or extremely high-power light sources (150 W halogen lamps).^73^ Taken together, these comparisons demonstrate that Z45 achieves top-tier solar-driven performance and robust UV responsiveness while completely bypassing high-temperature thermal treatments (500–700 °C), high-pressure autoclaves, and organic additives, underscoring its superior energy efficiency and sustainability footprint.

**Table 4:** Comparison of the photocatalytic efficiency of sample Z45 with reported pure ZnO catalysts for methylene blue (MB) degradation under solar and UV light irradiation

Catalyst (light)	Synthesis conditions	ZnO dosage-MB conc.	Max. efficiency (time)
Z45 (Sun), this work	Ambient conditions	1.0 g L^−1^–10 ppm	99.2% (150 min)
ZnO (Sun)^69^	Sol–gel (60 °C, calcination at 500 °C for 2 h)	2 g L^−1^–10 ppm	100% (180 min)
ZnO (Sun)^70^	Mechanochemical–calcination (500 °C, 3 h)	0.5 g L^−1^–26 ppm	68.70% (60 min)
Z45, (UV-A, 365 nm, 6 W), this work	Ambient conditions	1.0 g L^−1^–10 ppm	77.0% (150 min)
ZnO (UV-A 300–400 nm, 8 W)^71^	Sol–gel (130 °C, high pressure, calcination at 700 °C for 4 h)	1.45 g L^−1^–10 ppm	72.3% (420 min)
ZnO (UV-lamp, 11 W)^72^	Sol–gel (60 °C for 1 h, calcination 500 °C for 2 h)	0.25 g L^−1^–5 ppm	91.0% (180 min)
ZnO (W halogen lamp, 150 W)^73^	Precipitation, calcination at 500 °C for 3 h	0.2 g L^−1^–10 ppm	74.0% (80 min)

#### 3.4.3 Photocatalytic mechanism

To account for the high efficiency observed under sunlight (>99.2% MB removal within 150 min) as well as the UV-driven performance, a plausible reaction mechanism is proposed based on charge carrier generation and surface dynamics.

Upon light absorption (UV or solar radiation), electrons (e^−^) are promoted from the valence band (VB) to the conduction band (CB) of ZnO, generating electron–hole pairs (e^−^/h^+^):^68^

14 ZnO + *hν* → ZnO(e_CB_^−^ + h_VB_^+^)

Under UV light, direct band-gap excitation dominates. However, as demonstrated by our temperature-dependence study, the overall degradation rate is strongly bottlenecked by physical adsorption–desorption steps and charge recombination rates rather than energy barrier.^68,75^ The higher specific surface area of Z45 sample plays a key role in hosting a larger amount of absorbed MB molecules, maximizing the interaction with photogenerated reactive species before recombination occurs.^67^

The photogenerated charges react with adsorbed species to form the following primary active oxygen species (ROS):^75^

(1) Electrons in the CB reduce dissolved oxygen molecules to superoxide radicals:

15 e_CB_^−^ + O_2_ → ˙O_2_^−^

(2) Holes in the VB oxidize surface-bound water or hydroxyl ions into highly reactive radicals:16
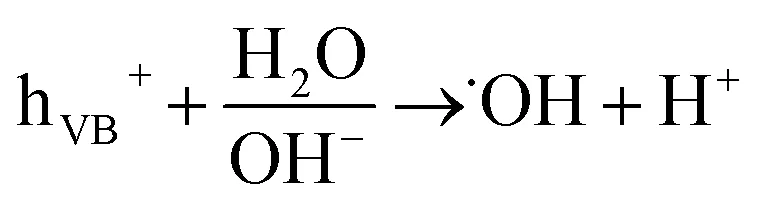


These generated radicals (˙OH and ˙O_2_^−^) non-selectively attack the methylene blue (MB) molecules, degrading them into smaller harmless intermediates, and ultimately achieving complete mineralization into CO_2_ and H_2_O.^75^

### 3.5 Thermodynamics and kinetic activation parameters

The kinetic plots of ln(C_0_/*C*_*t*_) *versus* time for methylene blue (MB) degradation under both artificial UV irradiation and natural solar illumination are shown in Fig. 8. The apparent rate constants (*k*_ap_) were derived from the slope of the linear regression curves according to eqn (7). The calculated *k*_ap_ values alongside their corresponding determination coefficients (*R*^2^) are summarized in Table 5. High determination coefficients (*R*^2^ > 0.96) were obtained across all experimental conditions, confirming that the photocatalytic degradation of MB strictly obeys pseudo first-order kinetic within the Langmuir–Hinshelwood framework.

**Fig. 8:**
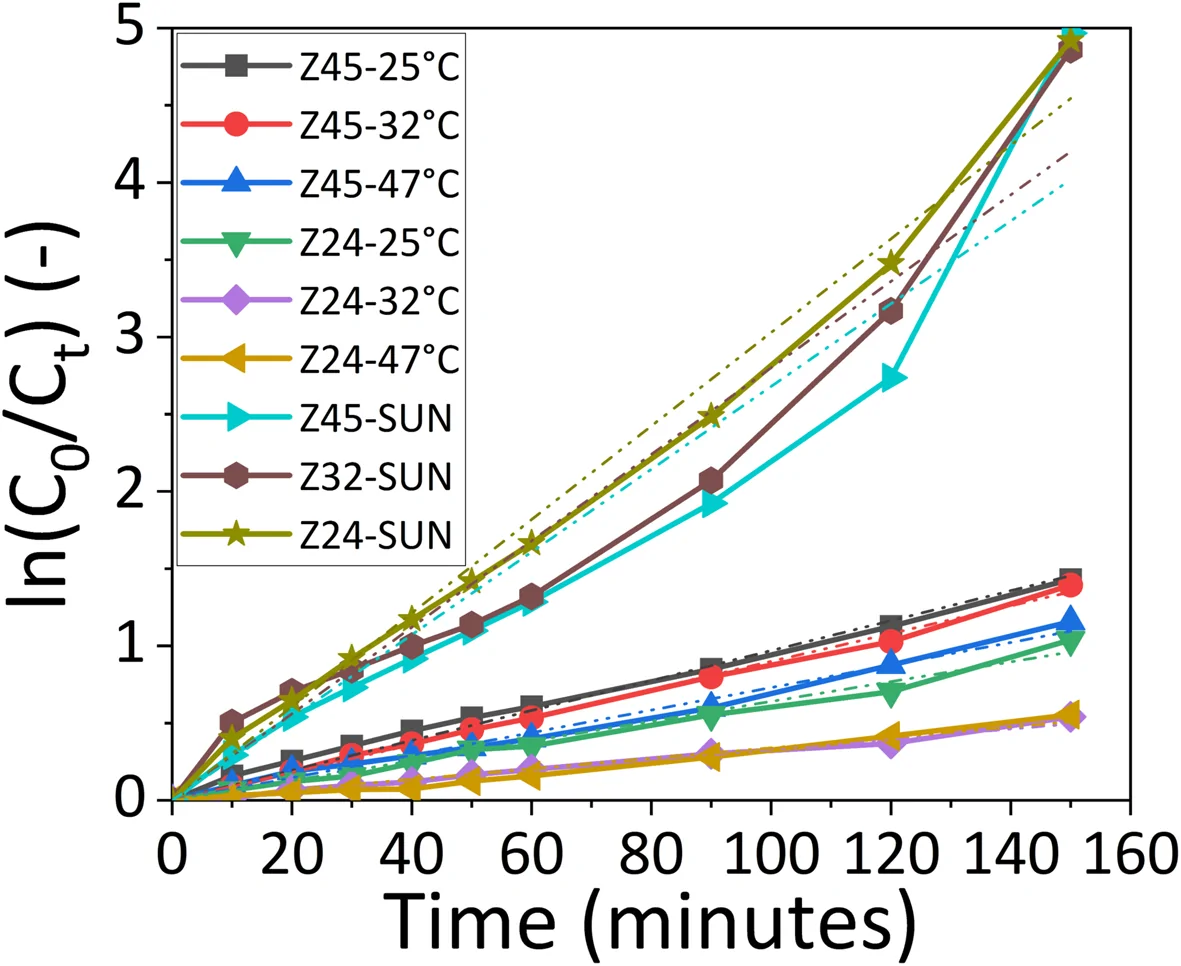
Linear fit of ln(C_0_/*C*_*t*_) *vs.* time for MB degradation under sunlight (Z45-Sun, Z32-Sun and Z24-Sun) and indoor with a 365 nm UV light at different temperatures (25, 32 and 47 °C).

**Table 5:** Summary of the apparent rate constants (*k*_ap_) and determination coefficients (*R*^2^) derived for MB degradation under indoor UV irradiation at different temperatures and under natural solar illumination (Sun)

Sample	*k*_ap_ (25 °C) UV (min^−1^)	*k*_ap_ (32 °C) UV (min^−1^)	*k*_ap_ (47 °C) UV (min^−1^)	*k*_ap_ (Sun) (min^−1^)
Z24	6.371 × 10^−3^	3.334 × 10^−3^	3.357 × 10^−3^	3.033 × 10^−2^
	(*R*^2^ = 0.994)	(*R*^2^ = 0.995)	(*R*^2^ = 0.980)	(*R*^2^ = 0.995)
Z32	—	—	—	2.803 × 10^−2^
				(*R*^2^ = 0.978)
Z45	9.692 × 10^−3^	9.001 × 10^−3^	7.336 × 10^−3^	2.682 × 10^−2^
	(*R*^2^ = 0.996)	(*R*^2^ = 0.999)	(*R*^2^ = 0.996)	(*R*^2^ = 0.962)

To gain insight into the energy barriers of the photo-elimination process, the temperature dependence of *k*_ap_ was investigated to evaluate the apparent activation energy (*E*_ap_), activation enthalpy (Δ*H*), and activation entropy (Δ*S*). Fig. 9 displays the Arrhenius plots (ln *k*_ap_*vs.* 1/*T*) and Eyring plots (ln(*k*_ap_/*T*) *vs.* 1/*T*):

**Fig. 9:**
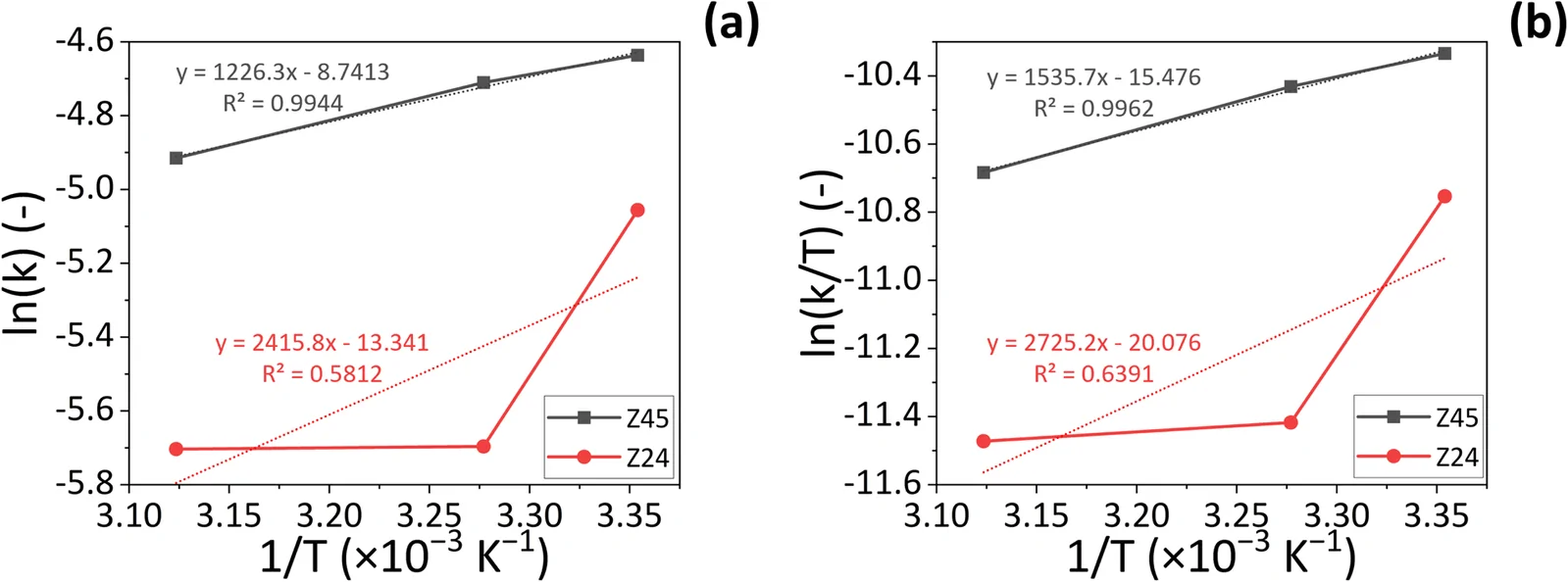
Arrhenius plot (a) and Eyring plot (b) of Z24 and Z45 samples.

Linear regression of the Arrhenius and Eyring plots yielded the following kinetic and thermodynamic parameters:

– Apparent activation energy (*E*_ap_): −10.20 kJ mol^−1^ for Z45 and −20.09 kJ mol^−1^ for Z24;

– Activation enthalpy (Δ*H*): −12.77 kJ mol^−1^ for Z45 and −22.66 kJ mol^−1^ for Z24;

– Activation entropy (Δ*S*): −330 J (mol^−1^ K) for Z45 and −360 kJ (mol^−1^ K) for Z24.

In addition, the Gibbs free energy of activation (Δ*G*) was calculated at each temperature (298.15 K, 305.15 K, and 320.15 K) and is illustrated in Fig. 10.

**Fig. 10:**
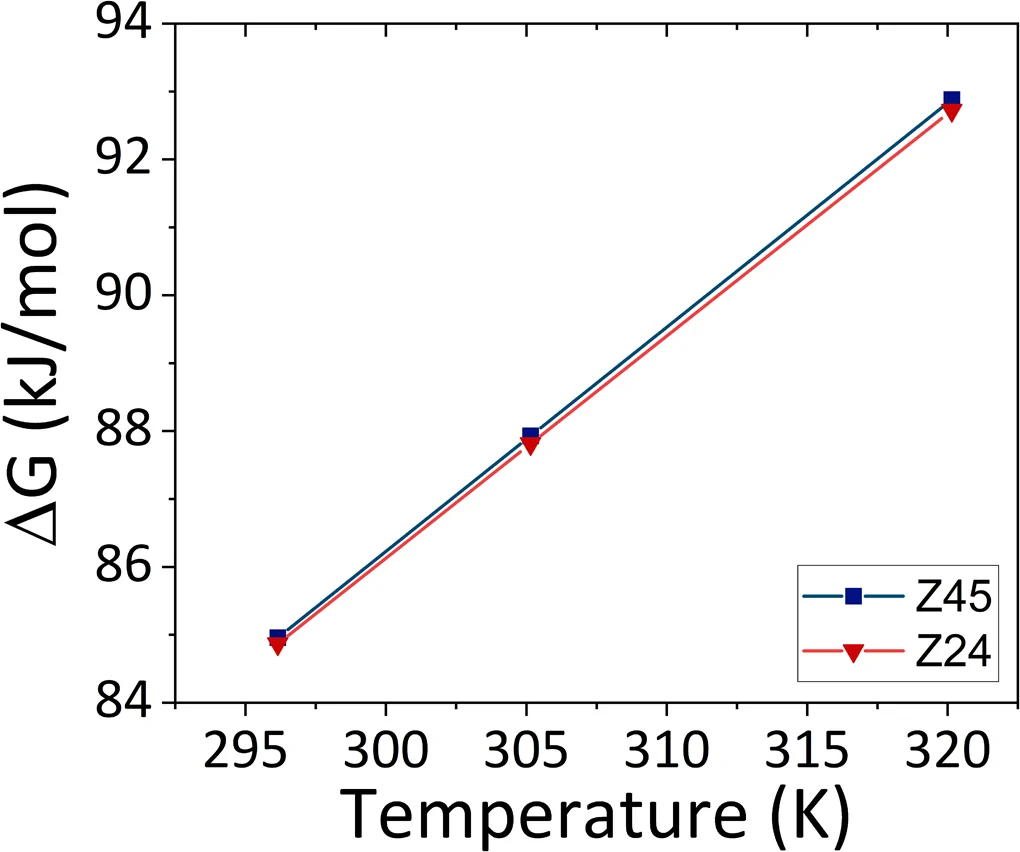
Gibbs free energy of activation ((Δ*G*) for Z24 and Z45 at 298.15 K, 305.15 K and 320.15 K.

The positive values of Δ*G* reflect the endergonic nature of the transition state formation during the photocatalytic process. Notably, Z24 exhibits a lower Gibbs energy barrier compared to Z45, which quantitively supports the superior overall photocatalytic efficiency of Z24. The negative entropy (Δ*S* < 0) for both samples further indicates an associative mechanism, wherein the rotational and translational degrees of freedom of the MB molecules and intermediate species are restricted upon adsorption onto the active surface.

While positive Δ*G* values confirm the endergonic character of the transition state, a closer inspection of the temperature dependence reveals a negative apparent activation energy (*E*_ap_ < 0) for both catalysts. This reflects the observed decrease in degradation rate at high temperatures, a behaviour characteristic of a non-Arrhenius kinetic regime. In this regime, exothermic adsorption–desorption equilibria and temperature-induced charge carrier recombination dominate over the thermally activated elementary reaction steps.

In contrast, the true intrinsic activation energy (*E*_a_) remained virtually identical for both samples (2.57 kJ mol^−1^). The occurrence of a negative *E*_ap_ strongly indicates that the rate-determining step is preceded by a highly exothermic physical/chemical adsorption equilibrium, a phenomenon well-documented in heterogeneous photocatalysis operating under the Langmuir–Hinshelwood framework.^76,77^

While *E*_ap_ reflects the global temperature response, conflating the adsorption enthalpy (Δ*H*_ads_) and the intrinsic activation energy (*E*_a_), the invariance of *E*_a_ across samples confirms that the fundamental chemical pathway for MB mineralization is unaffected by the synthesis conditions.^78,79^ Consequently, the observed divergence in *E*_ap_ values can be unequivocally ascribed to differences in surface-pollutant affinity (Δ*H*_ads_), which are modulated by the specific crystallographic facets and active surface area exposed by each sample.^80,81^

### 3.6 Fourier-transform infrared (FTIR) analysis and catalyst stability

Attenuated Total Reflection Fourier-Transform Infrared spectroscopy (ATR-FTIR) was employed to investigate the chemical structure, surface functional groups and structural stability of the synthesized nanoparticles, before and after photocatalytic methylene blue (MB) degradation (Fig. 11).

**Fig. 11:**
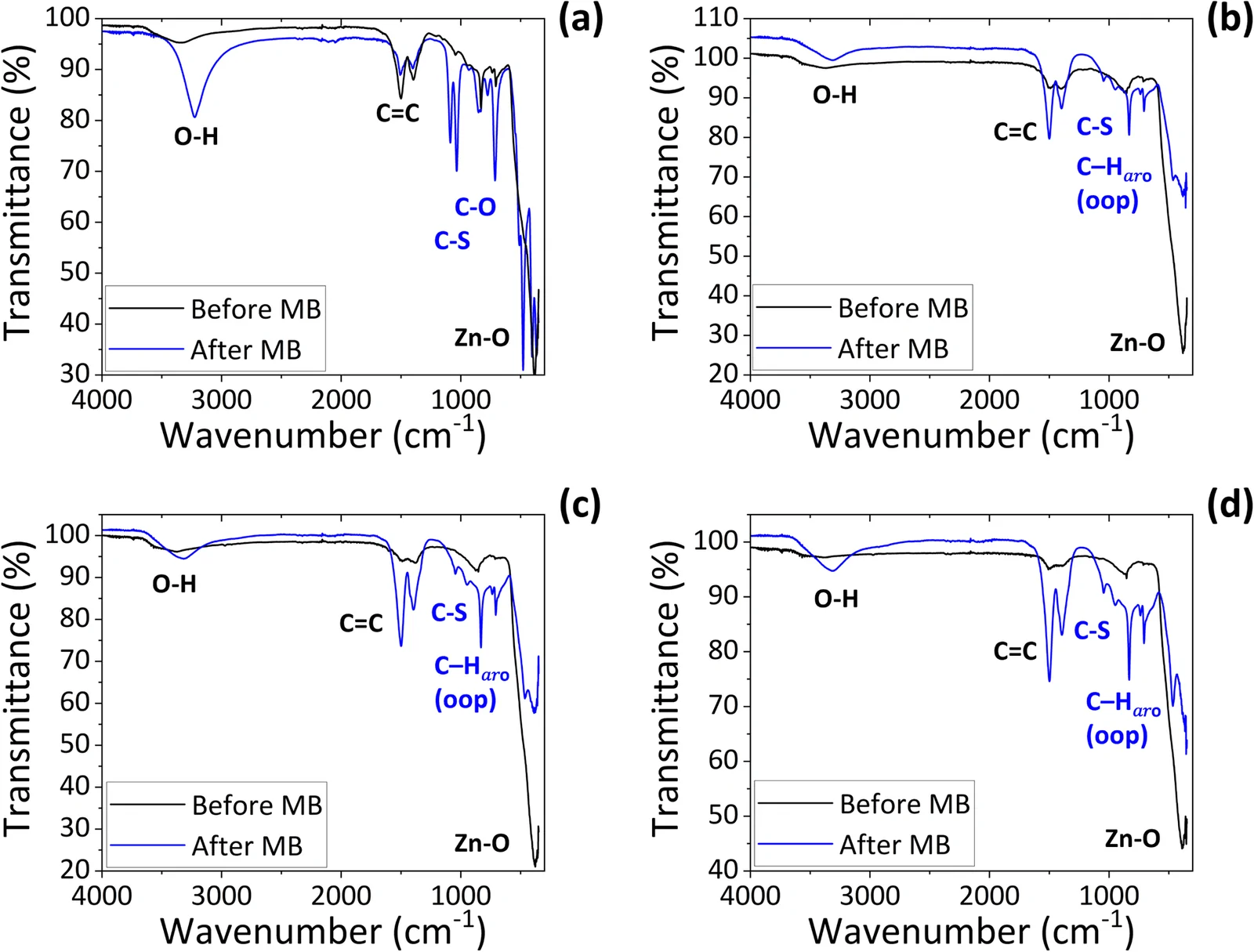
FTIR analysis before MB degradation (black curves) and after MB degradation (blue curves) of Z15 (a), Z24 (b), Z32 (c), and Z45 (d).

For the fresh samples synthesized at 24, 32 and 45 °C (Fig. 11b–d, black curves), characteristic absorption bands are observed at approximately 3400 cm^−1^, 1500 cm^−1^ and 390 cm^−1^. These bands correspond to the O–H stretching vibrations of surface-adsorbed water, C=C bending modes, and the fundamental Zn–O lattice stretching vibration, respectively.^82,83^ For the sample synthesized at 15 °C (Fig. 11a)), an additional distinct peak appears at approximately 490 cm^−1^, assigned to Zn–O–H bending vibrations. This feature reflects a higher extent of surface hydroxylation and confirms the predominant presence of the ε-Zn(OH)_2_ phase at lower synthesis temperatures.

A closer inspection of the spent catalysts (blue curves) reveals key mechanistic insights into the surface-driven degradation process. For the pure ZnO samples (Z45, Z32, and Z24), new absorption modes emerge at approximately 1000 cm^−1^ (C–S stretching) and 960 cm^−1^ (out-of-plane aromatic C–H bending). Remarkably, the intensity of these intermediate bands follows the trend Z45 > Z32 > Z24, directly reflecting the order of specific surface area and photocatalytic degradation rate. In Z45, the higher surface area (35.76 m^2^ g^−1^) and abundant defect states facilitate an intensive surface-mediated ROS attack, leading to a higher concentration of transient aromatic fragments (resulting from the cleavage of the MB central heterocycle) adsorbed on the active sites.

Conversely, for the sample synthesized at 15 °C (Z15, Fig. 11a)), the post-reaction spectrum displays a significantly intensified and broadened –OH stretching band (3400 cm^−1^), alongside sharp bands corresponding to C–S and C–O stretching modes. This behaviour is attributed to the hydroxyl-rich nature of the ε-Zn(OH)_2_ matrix, which promotes extensive surface hydration and traps partially oxidized, oxygenated degradation intermediates (containing C–O bonds).

Overall, despite the adsorption of these reaction intermediates, the fundamental inorganic core maintains complete structural stability during operations. The retention of Zn–O lattice vibration (≈390 cm^−1^) across all spent samples confirms that the dye breakdown occurs *via* surface-mediated ROS attack without inducing structural breakdown or photocorrosion of the ZnO matrix.^84^

### 3.7 Optical bandgap analysis

The optical absorption properties of the synthesized ZnO nanoparticles (Z45, Z32, and Z24) were investigated using UV-vis spectroscopy. Fig. 12 displays the optical absorption spectra (absorbance *vs.* wavelength) in the range of 200–800 nm. All samples exhibit a distinct, sharp absorption edge in the UV region 350–380 nm, which is characteristic of the intrinsic band-to-band electronic transitions in broad bandgap ZnO. Minimal absorption is observed across the visible spectrum (400–800 nm), confirming the high structural quality and phase purity of the nanoparticles.

**Fig. 12:**
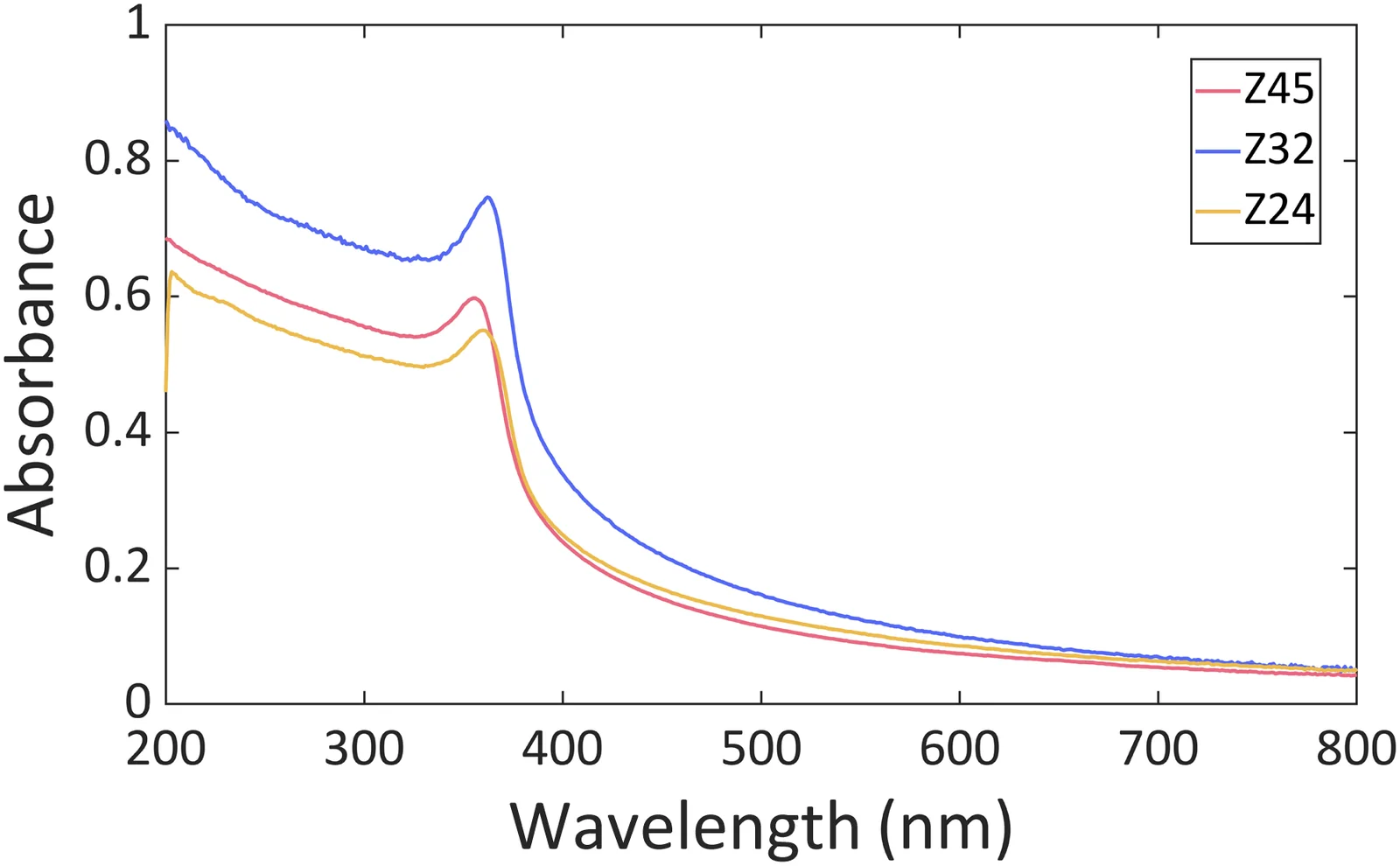
Absorbance spectrum from 200 to 800 nm for Z24, Z32 and Z45.

The optical band gap (*E*_g_) of the samples was subsequently determined using the Tauc plot method (eqn (12)).^58^ Extrapolation of the linear regime of the (*αhν*)^2^*vs. hν* curves yielded *E*_g_ value of 3.21 eV for Z24 and Z32, and 3.27 eV for Z45 (Fig. 13). The observed reduction compared to the standard bulk ZnO bandgap (3.37 eV)^50^ suggests a meaningful modification in the electronic band structure. Rather than quantum confinement, which typically induces a blue-shift in nanoscale structures, this bandgap narrowing points to enhanced structural disorder and the formation of localized defect states within the band gap. These shallow donor levels, likely originating from oxygen vacancies, facilitate sub-bandgap photo-excitation pathways.^82^

**Fig. 13:**
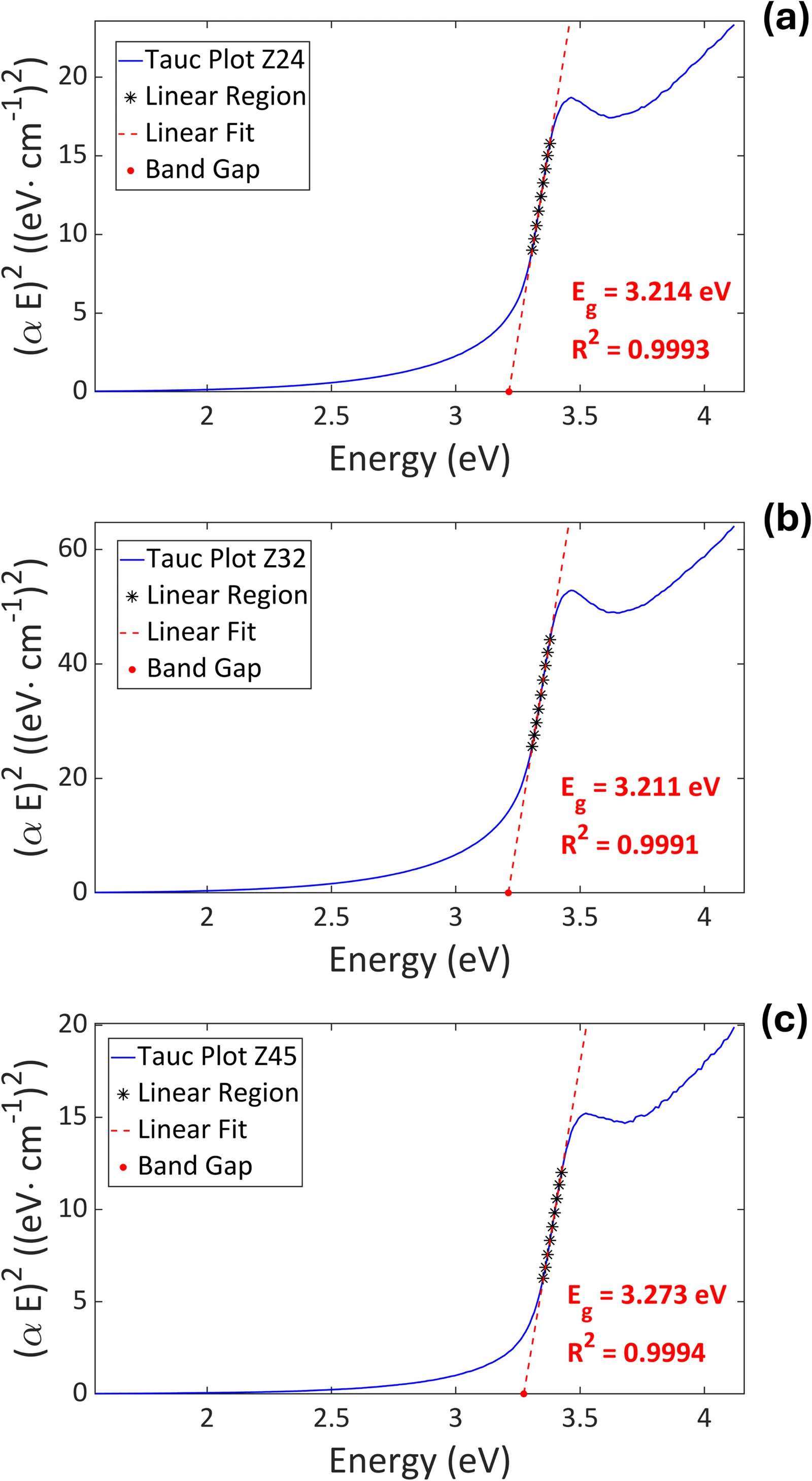
Band gap determination *via* Tauc plot for (a) Z24 (3.21 eV), (b) Z32 (3.21 eV) and (c) Z45 (3.27 eV).

The practical implications of this bandgap narrowing (*E*_g_ ≈ 3.21–3.27 eV) are directly reflected in the photocatalytic degradation of methylene blue (MB). Remarkably, the maximum degradation efficiency was significantly higher under natural solar irradiation (99.2%) compared to indoor monochromatic UV illumination at 365 nm (≈80%). While 365 nm photons (approx. 3.40 eV) possess sufficient energy to bridge the fundamental bandgap and initiate the photocatalytic process, the standard indoor UV lamp delivers a relatively low and strictly monochromatic photon flux. In contrast, the superior performance under sunlight stems from a synergistic exploitation of the broader solar spectrum. Intrinsic structural defects and oxygen vacancies introduce localized intermediate states that extend the light-harvesting capacity of the ZnO into the visible domain, enabling the material to harvest a much larger fraction of the solar photon flux to generate reactive oxygen species (ROS).^82,83^

Furthermore, to evaluate the thermodynamic feasibility of ROS generation over the synthesized ZnO nanoparticles, the conduction band (*E*_CB_) and valence band (*E*_VB_) edge potentials were calculated using the Mulliken electronegativity theory:^72^

17 *E*_VB_ = *χ* − *E*_e_ + 0.5*E*_g_

18 *E*_CB_ = *E*_VB_ − *E*_g_

where χ is the Mulliken absolute electronegativity of ZnO (5.47 eV),^74^*E*_e_ is the energy of free electrons on the hydrogen scale, which is 4.5 eV relative to the Normal Hydrogen Electrode (NHE), and *E*_g_ is the calculated optical band gap, (3.27 eV for Z45, 3.21 eV for Z32 and Z24). Based on the eqn (17) and (18), the calculated band positions *vs.* NHE are:*E*_CB_ = −0.345 V and *E*_VB_ = +2.925 V (Z45)*E*_CB_ = −0.315 V and *E*_VB_ = +2.895 V (Z32 e Z24)

For all samples, the *E*_CB_ potentials are more negative than the standard redox potential for single-electron oxygen reduction (*E*^0^(O_2_/˙O_2_^−^) = −0.16 V *vs.* NHE), confirming that photogenerated electrons (e^−^) can effectively reduce dissolved O_2_ to superoxides radicals (˙O_2_^−^). Simultaneously, the *E*_VB_ potentials are significantly more positive than the oxidation potential of H_2_O/˙OH (*E*^0^(H_2_O/˙OH) = +2.38 V *vs.* NHE), indicating that photogenerated holes (h^+^) can directly oxidize surface-adsorbed species to yield highly reactive hydroxyl (˙OH) radicals.

## 4 Conclusions

In summary, this work presents a sustainable, additive-free ion-exchange strategy for the rapid, ambient synthesis of ZnO and ε-Zn(OH)_2_ nanostructures. Crucially, ordinary tap water acts as an active chemical mediator, bypassing energy-intensive calcination and distilled water usage. Temperature was identified as the key thermodynamic lever governing phase selection, driving a structural transition from ε-Zn(OH)_2_ at 15 °C to pseudo-spherical ZnO nanoparticles (≈9 nm, 35.76 m^2^ g^−1^) within just 2 minutes at 45 °C.

All the photocatalysts demonstrated exceptional solar-driven activity, achieving >99.2% methylene blue degradation within 150 minutes under natural sunlight. Defect-induced bandgap narrowing (*E*_g_ ≈ 3.1–3.27 eV) and oxygen vacancies expand its light-harvesting capacity into the solar spectrum. Benchmarking against representative pure ZnO catalysts in the literature underscores that Z45 achieves superior or comparable solar degradation rates without requiring energy-intensive thermal calcination (500–700 °C) or high-pressure autoclaves.

Mechanistically, the reaction follows non-Arrhenius kinetics with a negative apparent activation energy (*E*_ap_), confirming that degradation is governed by favourable exothermic adsorption equilibria. Furthermore, post-reaction FTIR analysis confirmed the high structural stability and resistance to photocorrosion of the synthesized ZnO framework. Overall, this approach successfully connects green synthesis parameters with thermodynamic insight, providing a scalable pathway for solar-driven environmental remediation.

## Author contributions

Conceptualization and methodology: G. T., O. S.; investigation and data curation: G. T., V. M., M. C.; writing – original draft preparation: G. T., V. M., M. C.; validation and visualization: G. T., V. M., M. C., V. D., C. M., O. S.; formal analysis and editing: G. T., V. M., M. C.; supervision: G. T., C. M., O. S. All authors have read and agreed to the published version of the manuscript reported.

## Conflicts of interest

There are no conflicts to declare.

## Data Availability

The original contributions presented in this study are included in the article. Further inquiries can be directed to the corresponding author.
